# Phage isolation and functional characterization reveal strong antibiofilm activity against *Pseudomonas aeruginosa* in a cystic fibrosis sputum model

**DOI:** 10.3389/fcimb.2026.1753740

**Published:** 2026-02-13

**Authors:** Madeline Bowder, Hannah Kapoor, Steven J Carrell, Lia Danelishvili

**Affiliations:** 1 Department of Microbiology, College of Science, Oregon State University, Corvallis, OR, United States; 2 Department of Biochemistry & Molecular Biology, College of Science, Oregon State University, Corvallis, OR, United States; 3 Center for Quantitative Life Sciences, Oregon State University, Corvallis, OR, United States; 4 Department of Biomedical Sciences, Carlson College of Veterinary Medicine, Oregon State University, Corvallis, OR, United States

**Keywords:** A549 epithelial cells, biofilm, colonization, jumbo phages, lytic phages, phage therapy, Pseudomonas aeruginosa

## Abstract

*Pseudomonas aeruginosa* is an opportunistic pathogen that forms persistent biofilms in the lungs of cystic fibrosis and other chronic pulmonary disease patients, contributing to antibiotic tolerance, recurrent infection, and clinical decline. The rise of multidrug-resistant *P. aeruginosa* underscores the urgent need for alternative therapies. Bacteriophages (phages) offer a powerful therapeutic approach by directly lysing bacteria, diminishing biofilm structures, and overcoming mechanisms that limit antibiotic efficacy. In this study, a library of 61 distinct *P. aeruginosa* phages was isolated and screened against 64 clinical isolates, identifying eight with broad host range and high lytic activity. These phages, including PA-319, PA-575, and PA-711, effectively prevented *P. aeruginosa* colonization on A549 human lung epithelial cells, inhibited bacterial biofilm formation as well as compromised established biofilms, surpassing the effects of high-concentration antibiotics. Genomic and transmission electron microscopy analyses revealed functional heterogeneity, including nucleus-forming and non-nucleus-forming jumbo phages and depolymerase-encoding genes. Our phage library provides a valuable resource for advancing research, developing combinatorial phage therapies, and optimizing treatment strategies against chronic, drug-resistant *P. aeruginosa* infections.

## Introduction

*Pseudomonas aeruginosa* is a gram negative, rod shaped, motile bacterium (Ambreetha and Singh, 2023) with a relatively large (5.5–7 Mbp) genome, which encodes a broad range of virulence genes (Pang et al., 2019). This extensive coding capacity enables the bacteria to be highly ubiquitous and adaptable, contributing to its ability to thrive in a variety of hosts including nematodes, insects, reptiles, plants, animals, and humans (Ambreetha and Singh, 2023). The facultative anaerobic lifestyle further supports survival and colonization of *P. aeruginosa* in diverse ecological spaces, including soil, water, hospitals, and household sink drains (Tuon et al., 2022; Ambreetha and Singh, 2023).

As an opportunistic pathogen, *P. aeruginosa* rarely colonizes healthy individuals, but can cause life threatening infections in immunocompromised patients, such as those with pulmonary disease, cancer, AIDs, diabetes, wounds, and burns (Mulcahy et al., 2014). The ability of this pathogen to evade the innate and adaptive immune defenses contributes to its pathogenesis and clinical relevance (Tuon et al., 2022). *P. aeruginosa* is a leading cause of nosocomial infections (Jacobs et al., 2020), which are responsible for infections in 2 million patients annually and result in 90,000 deaths each year (Mulcahy et al., 2014). Healthcare-associated infections are often linked to colonization of medical devices such as ventilators, intravenous lines, catheters, medical implants, and endotracheal tubes, where biofilms readily form (Tuon et al., 2022). These biofilm infections pose grave threats to patient outcomes and create challenges to their clinicians (Mulcahy et al., 2014).

Biofilm formation is a key virulence factor in establishing persistent, chronic infections by *P. aeruginosa*, particularly in the lungs of patients with underlying pulmonary conditions such as cystic fibrosis (CF), bronchiectasis, and chronic obstructive pulmonary disease (COPD) (Jacobs et al., 2020). In CF, this pathogen is a significant contributor to the morbidity and mortality of patients, increasing their risk of death 2.6 times (Jacobs et al., 2020). By adolescence, up to 85% of CF patients suffer from chronic *P. aeruginosa* infections, which accelerate lung damage and progress respiratory decline (Davies, 2002). Similarly, 4% to 15% of people with COPD are colonized by *P. aeruginosa*, which increases risk of overall clinical decline. Patients with bronchiectasis are colonized with *P. aeruginosa* less frequently, but cases are more severe, increasing the risk of death 3-fold (Jacobs et al., 2020). These chronic pulmonary infections are notoriously difficult to eradicate. Even rigorous antibiotic regimens fail to completely eradicate infection in 10-40% of CF patients, underscoring the limitations of current antimicrobial agents and the need for novel therapies (Varannai et al., 2021).

Phages have re-emerged as promising therapeutic agents to address the antibiotics therapy failures in refractory, drug-resistant, and biofilm associated infections (Lin et al., 2017). Phages are bacterial predators which self-amplify, co-evolve alongside bacteria, and are highly specific to their target pathogen, providing key advantages over conventional antimicrobials (Lin et al., 2017). Notably, large number of lytic *P. aeruginosa* phages with anti-biofilm activity have been identified and their mechanisms have been characterized (Chegini et al., 2020). Phages demonstrate a remarkable capacity to infiltrate the biofilm matrix and reach the deeper strata, areas where antibiotics often fail to diffuse effectively (Chegini et al., 2020). In addition, phages can degrade or interfere with bacterial biofilm formation by encoding depolymerase enzymes or through quorum-sensing suppression (Chegini et al., 2020; Visnapuu et al., 2022). Substantial research shows that phages are powerful controllers of *P. aeruginosa* biofilm infections (Guo et al., 2019).

Furthermore, phages can exhibit synergistic interactions when co-administered with antibiotics, thereby mitigating inherent limitations such as restricted host range and the emergence of phage-resistant bacterial variants (Chegini et al., 2020). It is also established that bacterial evasion of phage predation often incurs fitness trade-offs, leading to attenuated virulence characterized by diminished biofilm formation, decreased antibiotic resistance, impaired type III secretion, and reduced motility or swarming capacity (Chan et al., 2016; García-Cruz et al., 2024). Recent studies with phage OMKO1 have shown that it targets the outer membrane protein OprM, a component of the MexAB and MexXY efflux pump systems of *P. aeruginosa*, thereby driving an evolutionary trade-off that enhanced antibiotic susceptibility in multidrug resistant (MDR) *P. aeruginosa* (Chan et al., 2016). Comparable virulence trade-offs have also been observed in phages that employ lipopolysaccharides (LPS) or type IV pili as receptor-binding sites (García-Cruz et al., 2024; Chan et al., 2025). Such phages decreased bacterial loads and improved lung function of CF patients colonized with MDR or pan-drug resistant *P. aeruginosa* in a compassionate care phage therapy study (Chan et al., 2025).

Beyond individual case successes, clinical studies have provided additional evidence supporting the *in vivo* use of *P. aeruginosa* phages. Their efficacy and safety are currently being evaluated in several ongoing trials (NCT05453578, NCT04684641, NCT05010577, and NCT04803708) which use intravenous, nebulized, or topical phage cocktails to treat *P. aeruginosa* infections in CF patient lungs and diabetic foot ulcers (National Institute of Allergy and Infectious Diseases (NIAID), 2025; Nir-Paz et al., 2025; Rappo et al., 2891). Findings from these clinical trials demonstrate the safety and therapeutic potential of phage therapy in managing drug-resistant *P. aeruginosa* infections.

Despite these advances, significant gaps remain in our understanding of phage biology and their limitations, which must be addressed before phages can be considered robust therapeutic candidates. Key challenges include their often limited host range, the potential for the emergence of bacterial resistance, concerns regarding phage-associated toxicity, and the need for clear regulatory frameworks governing phage therapy (Lin et al., 2022). The isolation and comprehensive characterization of diverse phage libraries is essential for overcoming challenges such as limited host range and bacterial escape. By enabling the development of tailored phage cocktails and informing synergistic use with antibiotics, these efforts directly contribute to the advancement of phage therapy, the objective that forms the central aim of this study.

## Materials and methods

### *P. aeruginosa* strains and culture conditions

*P. aeruginosa* reference strains PAO1 and PA14 strains were provided by Dr. Martin Schuster at College of Science, Oregon State University. A total of 64 P*. aeruginosa* isolates were used in this study, including 40 human clinical isolates and 24 animal isolates. Human isolates of *P. aeruginosa* were obtained from the Biodefense and Emerging Infections Research Resources Repository (BEI Resources), and animal isolates were sourced from the Carlson College of Veterinary Medicine (CCVM) Bacteriology Diagnostic Laboratory at OSU. Isolates were selected to represent diverse antimicrobial susceptibility profiles and a range of clinical sources (**Supplementary Tables 1-3**). Bacterial stocks were preserved in Luria-Bertani (LB) broth supplemented with 50% glycerol (1:1, v/v) and stored at –80°C. For experimental use, bacteria were cultured as needed on LB agar plates or in LB broth.

### Antibiotic susceptibility and minimum inhibitory concentration

Antibiotic susceptibility data for clinical isolates were provided by BEI Resources and OSU CCVM Diagnostic Laboratory. Amikacin and ciprofloxacin were purchased from Sigma-Aldrich. The MIC of PAO1 was determined by the broth microdilution according to the guidelines of the European Society of Clinical Microbiology and Infectious Diseases (European Committee for Antimicrobial Susceptibility Testing (EUCAST) of the European Society of Clinical Microbiology and Infectious Diseases (ESCMID), 2003). Briefly, LB broth (4 mL) was serially diluted two-fold to generate antibiotic concentrations ranging from 256 μg/mL to 0.25 μg/mL. The bacterial inoculum was prepared in Hank’s Balanced Salt Solution (HBSS) and standardized to a 1.0 McFarland standard, diluted to a final concentration of 1×10^5^ colony forming units (CFU)/mL and inoculated into each antibiotic-containing dilution. Cultures were incubated at 37 °C with shaking for 24 hours. The MIC was defined as the lowest antibiotic concentration that prevented visible bacterial growth. All experiments were performed in biological triplicates.

### Environmental sampling and filtrate preparation for phage isolation

The environmental samples used in this study were originally collected for the isolation of *Mycobacterium avium* subsp. *paratuberculosis* phages, as previously described in Golla et al, 2024 (Golla et al., 2024). Briefly, over 900 environmental samples (water, soil, and soil–manure mixture) were collected from dairy farms in Asia, North America, and South America. Solid samples were suspended in phage buffer (10 mL 1 M Tris/HCl, pH 7.5; 10 mL 1 M MgSO_4_; 4 g NaCl; 980 mL H_2_O) and gently agitated for 24 hours. Suspensions were centrifuged, and the supernatants were sequentially filtered through 1.0 μm, 0.45 μm, and 0.2 μm syringe filters. Filtrates were stored at 4 °C until use.

### Generation of *P. aeruginosa* phage library

The reference *P. aeruginosa* strain PAO1 was grown overnight on LB agar plates at 37 °C. Bacterial inoculum was prepared by suspending PAO1 colonies in phage buffer, briefly vortexing, sonicating for 1 min, and adjusting the suspension to 3×10^8^ CFU/mL (1.0 McFarland standard). Aliquots of 100 μL of the bacterial inoculum were mixed with 300 μL of environmental sample filtrates, vortexed, and incubated overnight at room temperature. The mixtures were then combined with 4 mL of LB top agar (0.6% Bacto Agar, 4.7 g/L LB broth, 100 mL deionized water) and overlaid onto LB agar plates. Plates were incubated overnight at 37 °C.

Plaques were excised using sterile transfer pipettes, suspended in phage buffer, and stored at 4 °C overnight. For propagation, individual plaques were diluted and re-infected into fresh PAO1 cultures by titration using the top agar overlay method. High-titer phage stocks were generated through re-propagation utilizing the “webbing pattern” technique. Phages were collected by overlaying plates with 4 mL of phage buffer, followed by centrifugation of supernatants at 8,000 x *g* for 10 minutes and filtration through a 0.2 μm polyethersulfone membrane filter. Final phage lysates were quantified by titration using the double-layer agar method, yielding titers ≥10^10^ PFU/mL, and were stored at 4 °C.

### Phage host range

Phage host range against PA14 and 64 P*. aeruginosa* clinical isolates was assessed by spot testing. *P. aeruginosa* inoculum was prepared in HBSS, adjusted to a 1.0 McFarland standard, and 100 μL aliquots were mixed with 15 mL LB top agar and overlaid onto LB agar prepared in 120 × 120 mm Corning square plates. A library of 61 P*. aeruginosa* phages was arranged in a 96-well plate, and 4 μL aliquots of each phage (10^10^ PFU/mL) were spot-plated on bacterial lawns. Plates were incubated overnight at 37 °C. A clear lysis zone was considered indicative of phage susceptibility.

### Phage efficiency of plating

To evaluate the potency of phage activity against *P. aeruginosa* isolates, an EOP assay was performed using the eight phages with the broadest host ranges, capable of lysing ≥48 of 65 strains (PA14 and 64 clinical isolates). The reference strain PA14, along with seven clinical isolates representing diverse host species, clinical sources, and varying levels of antimicrobial resistance, were included to assess both the EOP and the potential clinical applicability of these phages. Phages were serially diluted ten-fold in phage buffer and arranged in a 96-well plate. Bacterial cultures were prepared in HBSS, adjusted to a 1.0 McFarland standard, and mixed with 15 mL of top agar, which was then overlaid onto square LB agar plates. Aliquots (4 μL) of phage dilutions from the 96-well plate were spot-plated on the clinical isolate lawns, alongside the PAO1 reference strain. Assays were performed in triplicate. EOP values were quantified as the ratio of the titer of phage at the terminal dilution on the clinical isolate to the titer of the same phage on PAO1.

### Phage efficacy against biofilms

The anti-biofilm activity of selected phages was assessed under two conditions: inhibition of biofilm formation (pre-treatment) and disruption of established biofilms (post-treatment). PAO1 biofilms were generated in synthetic cystic fibrosis sputum medium (SCFM) as previously described by other groups (Palmer et al., 2007).

For the pre-biofilm conditions, phages were diluted in SCFM to 1×10^9^ PFU/mL and 100 μL aliquots were added to the experimental wells of round-bottom 96-well plates. Wells containing 100 μL amikacin (100 μg/mL) or ciprofloxacin (50 μg/mL) diluted in SCFM served as negative controls, and untreated SCFM wells served as positive controls for bacterial growth. PAO1 inocula were adjusted to a 1.0 McFarland at 3×10^8^ CFU/mL, diluted tenfold in SCFM, and 200 μL were added to each well. Plates were incubated at 25 °C for 24 h (eight technical replicate per treatment condition).

For the post-biofilm assays, 24-hour biofilms were established by incubating PAO1 (3×10^8^ CFU/mL, made in SCFM, 200 μL per well) for 24 h at 25 °C. Biofilms were then treated with 200 μL of phages (1×10^9^ PFU/mL), antibiotics, or SCFM and incubated for 24 h.

Biofilm biomass was quantified using crystal violet staining. Briefly, planktonic bacteria were gently aspirated to preserve biofilm integrity. The biofilms were washed once with Phosphate-Buffered Saline (PBS), then incubated with 200 μL of 0.1% crystal violet for 30 min at 25 °C. After staining, wells were washed once with 200 μL PBS, and the bound dye was solubilized with 200 μL of 30% acetic acid for 1 h on a rocker. Absorbance was measured at 590 nm using an Epoch Microplate Spectrophotometer (BioTek, Winooski, VT, USA) (Merritt et al., 2005). Both experiments were performed in three biological replicates.

### Quantification of bacterial viability in biofilms

Bacterial viability within pre- and post-treatment biofilms was quantified by measuring colony-forming units of viable bacteria in both the supernatants (planktonic bacteria) and the total bacterial population within each well. After biofilm formation and treatment, planktonic supernatants were removed and serially diluted in PBS. 4 μL of each dilution were spot plated on LB agar for CFU enumeration. For total bacterial counts, well contents were thoroughly resuspended by pipetting to disrupt the biofilm structure. The resulting suspension was serially diluted in PBS, and 4 μL aliquots of each dilution were spot plated onto LB agar plates. The number of biofilm-associated bacteria was determined by subtracting the CFUs of planktonic bacteria from the total bacterial count per well. Assays were repeated in three independent biological replicates.

Biofilms of MDR clinical isolates were tested alongside *P. aeruginosa* PAO1 and PA14 reference strains. Bacterial suspensions were adjusted to 3 × 10^7^ CFU/mL in SCFM, and 200 μL were added to round-bottom 96-well plates (12 technical replicates per condition). Following 48 hours of incubation at 37 °C, supernatants were removed, and biofilms were either treated with the selected phage PA-319 (1 × 10^9^ PFU/mL in SCFM) or replenished with SCFM alone as a control. After an additional 24-hour incubation at 37 °C, biofilms were stained with 0.1% crystal violet for 30 minutes, washed with PBS, and solubilized in 30% acetic acid for 1 hour. Absorbance was measured at 590 nm to quantify biofilm biomass. Experiments were performed in three independent biological replicates.

### Confocal microscopy of biofilms

PAO1 biofilms (made in SCFM, 3×10^7^ CFU/mL) were established in an 8-well chamber slide and incubated for 24 h at 37 °C (Filice et al., 2022). Supernatants were gently removed, and biofilms were either treated with selected phages (3 × 10^9^ PFU/mL) or left untreated, with SCFM added as a control, for an additional 24 hours. Biofilms were stained with SYTO^®^ 9 and propidium iodide using the FilmTracer™ LIVE/DEAD™ Biofilm Viability Kit (Invitrogen, USA) according to manufacturer’s protocol (Mountcastle et al., 2021). Stained biofilms were washed with sterile water, fixed with 2.5% glutaraldehyde for 1 hour, washed with PBS, and stored in PBS until imaging (Mountcastle et al., 2021). Confocal imaging was performed using a Zeiss LSM 780 NLO Confocal Microscope System at the OSU Center for Quantitative Life Sciences (CQLS) microscopy facility. Biofilms were imaged at 63x magnification using Z-stacks to capture the three-dimensional structure. Images were processed and examined with ImageJ for biofilm architecture, viability, and thickness.

### Tissue culture maintenance

The human lung carcinoma cell line A549 (CCL-185; ATCC) was cultured in Dulbecco’s Modified Eagle’s Medium (DMEM) and maintained in 75 cm^2^ flasks at 37 °C. Upon reaching 80-90% confluency, cells were treated with 3 mL TrypLE for 5 minutes (Chary, 2023), gently detached using a cell scraper, and centrifuged at 300 x *g* for 5 min. TrypLE was removed, and the cells were resuspended in DMEM. The cell concentrations were determined using a hemocytometer and 10^5^ cells for each well of 96-well plate was seeded.

### *P. aeruginosa* colonization of lung epithelial cells

A549 cells (1 × 10^5^ cells per well) were seeded into 96-well tissue culture plates and incubated at 37 °C with 5% CO_2_ until reaching 80–90% confluency. Cell monolayers were replenished with fresh media and pretreated with 10 µL of 1×10^9^ PFU/mL phage lysate (PA-312, PA-315, PA-319, PA-391, PA-394, PA-574, PA-575, or PA-711) for 30 minutes, with eight replicates for each treatment condition. Wells containing amikacin at the MIC or phage buffer served as controls. Cells were then infected with 100 µL of *P. aeruginosa* PAO1 inoculum (1 × 10^6^ CFU/mL, prepared in PBS and diluted16 in DMEM) per well, and plates were incubated for 6 hours at 37 °C (Carterson et al., 2005). Following incubation, both planktonic and colonized bacteria (intracellular or attached) were quantified using CFU counts for each treatment condition. Supernatants were carefully collected, serially diluted ten-fold in PBS, plated on LB agar, and incubated overnight at 37 °C. Cell monolayers were gently washed with PBS, lysed with sterile deionized water for 30 minutes, serially diluted, and plated on LB agar to determine viable bacterial counts. All experiments were conducted in three independent biological replicates.

### Scanning Electron (SEM) and Transmission Electron Microscopy

The scanning electron microscopy (SEM) was performed to examine the effect of phage PA-319 on *P. aeruginosa* colonization of A549 cells. This procedure was performed as described above except that monolayers were established on round coverslips. The tissue culture coverslips (15 mm diameter) were placed in 12-well plates, and 1.5 mL of A549 cells (1 × 10^6^ cells/mL) was added to each well. After incubation at 37 °C for 3 days, cells reached 80–90% confluency. Media was replaced with 850 µL of fresh DMEM, and wells were pretreated with 50 µL of phage PA-319 (1 × 10^9^ PFU/mL) or phage buffer as a control, followed by incubation at 37 °C for 30 minutes. Subsequently, 100 µL of *P. aeruginosa* PAO1 inoculum (3 × 10^6^ CFU/mL) was added, and plates were incubated for 6 hours at 37 °C. Monolayers were then washed three times with PBS, fixed with 2.5% glutaraldehyde for 1.5 hours, and stored in PBS until imaging (Garcia-Medina et al., 2005). After fixing, cells were dehydrated with increasing concentrations of ethanol and critical point dried with the Tousimis Autosamdri-931 (Tousimis, Rockville, MD). Coverslips were mounted onto stubs and coated with gold and palladium. Micrographs were taken with the FEI Quanta 600 FEG scanning electron microscope (Thermo Fisher Scientific) at the Oregon State University Electron Microscopy Facility.

TEM was performed to visualize jumbo phages and to examine nucleus-like structures formed during *P. aeruginosa* infection. Phages PA-319, PA-575, and PA-711 were purified and tittered to 1 × 10^10^ PFU/mL. For phage morphology analysis, 5 µL of each phage suspension was applied to Formvar-carbon–coated copper grids (400 mesh) and allowed to adsorb for 1 minute. Grids were gently blotted, negatively stained with 2% uranyl acetate for 1 minute, gently blotted, and imaged. To visualize intracellular phage replication structures, PAO1 cultures were prepared in PBS, adjusted to a 1.0 McFarland standard, and infected with phages PA-319 or PA-575 at a multiplicity of infection (MOI) of 1:100 (Cobián Güemes et al., 2023). Samples were incubated for 1 hour at 37 °C with gentle agitation, then fixed with 2.5% glutaraldehyde for 1.5 hours, washed three times with PBS, and stored in PBS until further processing. Infected bacterial pellets were rinsed in cacodylate buffer, embedded into agarose, and rinsed in buffer again, then post-fixed in 1% osmium tetroxide with 0.8% potassium ferricyanide for 1.5 hours, rinsed in water, *En bloc* stained with 4% uranyl acetate in ethanol and dehydrated through a graded ethanol series (50%, 70%, 90%, and 100%). Samples were then infiltrated and embedded in epoxy resin. Ultrathin sections (~80 nm) were cut using a Leica ultramicrotome (Leica, Deerfield, IL), mounted on copper grids, and stained with uranyl acetate followed by lead citrate. Micrographs were acquired using the FEI Titan 80–200/ChemiSTEM Transmission Electron Microscope (Thermo Fisher Scientific) operating at 80 kV at the Oregon State University Electron Microscopy Facility.

### DNA extraction of phages

Phage DNA was extracted using the GenElute™ Bacterial Genomic DNA Kit (Sigma-Aldrich) following the manufacturer’s protocol. Purified phages (450 µL) were initially treated with 1 µL DNase and 1 µL RNase in 50 µL buffer to remove contaminating bacterial nucleic acids. After 1.5 hours of incubation at 37 °C, the DNase and RNase were inactivated with 10 µL of 0.5 M EDTA. Proteinase K (1.25 µL of 20 mg/mL) was then added to digest phage capsid proteins, followed by incubation at 56°C for 1.5 hours. Whole lysates were processed for DNA purification by adding 200 µL Lysis Solution C and incubating for 10 minutes at 55 °C, after which 200 µL of ethanol was added. Lysates were transferred to binding columns and centrifuged at 8,000 x *g* for 1 minute. The columns were washed twice: first with 500 µL Wash Solution 1 and then with 500 µL Wash Solution Concentrate. DNA was eluted by adding 50 µL Elution Solution to the columns, incubating for 5 minutes at room temperature, and centrifuging for 1 minute at 8,000 x *g*. Purified DNA was collected in a fresh tube, and concentrations were determined using a Nanodrop. DNA samples were stored at –20°C until sequencing (Jakočiūnė and Moodley, 2018).

### Whole genome assembly of selected phages

Whole-genome samples were prepared using the Nextera XT DNA Library Preparation Kit (Illumina). Next-generation sequencing of phages was performed on a single lane on the Illumina NextSeq 2000 platform at the Oregon State University Center for Quantitative Life. The 150 bp paired-end raw reads were filtered and trimmed with Fastp 0.24.0 (Chen et al., 2018). Reads were then assembled *de novo* into contigs with Velvet v1.2.10 (Zerbino and Birney, 2008) and Trinity v2.15.1 (Grabherr et al., 2011). Phage genomes were assembled from the *de novo* contigs within the Geneious (version R2024) software platform (Kearse et al., 2012) using a multi-iterative assembly approach to resolve gaps. The resulting whole genome sequences (WGS) were confirmed by aligned the trimmed reads to the assemble genomes with Bowtie2 (Langmead and Salzberg, 2012), and quality checks were run in Geneious.

### Phage genome annotation

Phage genomes were initially annotated using SPHAE (Papudeshi et al., 2025) and further analyzed with InterProScan v5.75-106.0, applying an e-value threshold of 1e–10 for the top hit and 1e–15 for the second-best hit (Blum et al., 2021). Results from both analyses were integrated using a custom Python script to provide additional domain-level annotations for hypothetical proteins (https://github.com/hanana2000/Jumbo_Phage_Annotate/tree/main/combineSPHAEinterpro_script). Annotated genomes were then screened for potential depolymerase genes using keyword searches and local BLAST analyses against a curated database of known depolymerases (available at https://github.com/hanana2000/Jumbo_Phage_Annotate/blob/main/depolymerase_faas_detection/README.md) with DIAMOND v0.9.19 (Buchfink et al., 2015). Top candidate depolymerase hits were aligned with reference sequences using MAFFT v7.505 (Knecht et al., 2020) to determine substitution numbers, percent identity, conserved and weak substitutions, and other alignment statistics (Katoh et al., 2002). Additionally, phage genomes were annotated independently using Pharokka (Galaxy Version 1.3.2+galaxy0) (Bouras et al., 2023) and visualized in SnapGene Viewer v8.2 (www.snapgene.com). Tail-associated proteins identified through Pharokka annotations were compiled and analyzed with DePP web v1.0.0 to predict putative depolymerase activity (Magill and Skvortsov, 2023). Finally, jumbo phage genomes were screened in SnapGene for nucleus-forming protein keywords (Harding et al., 2023).

### Phage alignment and visualization

Whole-genome BLASTn alignments were performed using the NCBI BLAST alignment tool with an expected threshold of 0.05. In addition, local tBLASTx comparisons between the *P. aeruginosa* jumbo phages PA-319 and PA-575 were conducted using a minimum hit length of 45 amino acids, 30% identity, and an e-value cutoff of 1e–3 (Altschul et al., 1990; Camacho et al., 2009). Visual alignments and genome maps were generated in EasyFig v2.2.5 (Sullivan et al., 2011) based on tBLASTx results and color-coded using a custom Python script (https://github.com/hanana2000/Jumbo_Phage_Annotate/tree/main/add_color_notes). Phage genomes were further visualized and analyzed using Proksee (Moreau-Marquis et al., 2008), which provided GC skew and GC content plots. A whole-genome phylogenetic tree was generated with VipTree v4.0 (release 2023-11-17) (Nishimura et al., 2017).

## Results

### Antimicrobial resistance profiles of *P. aeruginosa* clinical isolates

To establish and categorize *P. aeruginosa* clinical strains based on their antibiotic susceptibility profiles, antibiogram reports were initially analyzed using data provided by BEI Resources and the Oregon State University Carlson College of Veterinary Medicine Diagnostic Laboratory (**Supplementary Tables 1**, **2**). The antibiotics tested for human isolates fall into three major antimicrobial classes commonly recommended for the treatment of *P. aeruginosa* infections: β-lactams, aminoglycosides, and quinolones (Zakhour et al., 2022). In contrast, animal-derived isolates were tested against a broader and more variable panel of antibiotics depending on the animal host species, spanning multiple antimicrobial classes, including aminoglycoside, amphenicol, β-lactam, cephalosporin, diaminopyrimidine, macrolide, penicillin, quinolone, sulfonamide, and tetracycline. Among the fifty-two *P. aeruginosa* strains with available resistance profiles, ten were classified as MDR, defined as resistance to at least one agent in three or more antimicrobial classes, and one strain met the criteria for pan-drug resistance (PDR), characterized by non-susceptibility to all agents across all antimicrobial classes.

### Creation of a diverse phage library against *P. aeruginosa*

To generate a phage library targeting *P. aeruginosa*, filtrates of environmental samples previously collected from diverse geographical locations (Golla et al., 2024) were screened for phages active in PAO1 reference strain using the double-layer agar method. Testing over 900 filtrates resulted in the successful isolation of 61 P*. aeruginosa* phages that were further propagated to achieve stock titers of at least 1×10^9^ PFU/mL. Out of 61 isolates, 54 phages formed clear plaques on PAO1 strain with well-defined edges, indicative of a lytic lifestyle, whereas seven phages produced turbid plaque morphology, suggestive of lysogenic potential. The isolated phages were arrayed in a 96-well plate format to create a comprehensive working library for downstream characterization of the PAO1 phage collection.

### Phage screening identifies broad-spectrum candidates active against MDR *P. aeruginosa* strains

Host range is a critical factor when selecting phages for therapeutic applications, as it determines the breadth of bacterial strains a single phage can effectively target (Myers et al., 2023). To comprehensively assess host range and clinical relevance, 61 isolated phages were screened against reference strain PA14 and 64 P*. aeruginosa* clinical isolates. These isolates were derived from infections in five mammalian hosts (human, canine, bovine, caprine, and equine) and collected from 11 anatomical sites, including the respiratory tract, wounds, ears, urine, lungs, skin, eyes, surgical sites, gastrointestinal fluid, burns, and other body fluids (**Supplementary Table 3**). Using the double-layer agar method, phages were spot-tested, and the presence of clear lysis zones indicated successful bacterial killing. Varied numbers of clear plaques were observed depending on the clinical strain susceptibility to individual phages. In some cases, isolates appeared to carry prophages that were induced upon exposure to lytic phages (**Supplementary Table 3**), generating additional lysis patterns. These prophages were identified based on their small plaque size, uniform morphology, and occurrence in areas of the plate where no phages were directly spot-plated.

**Figure 1:**
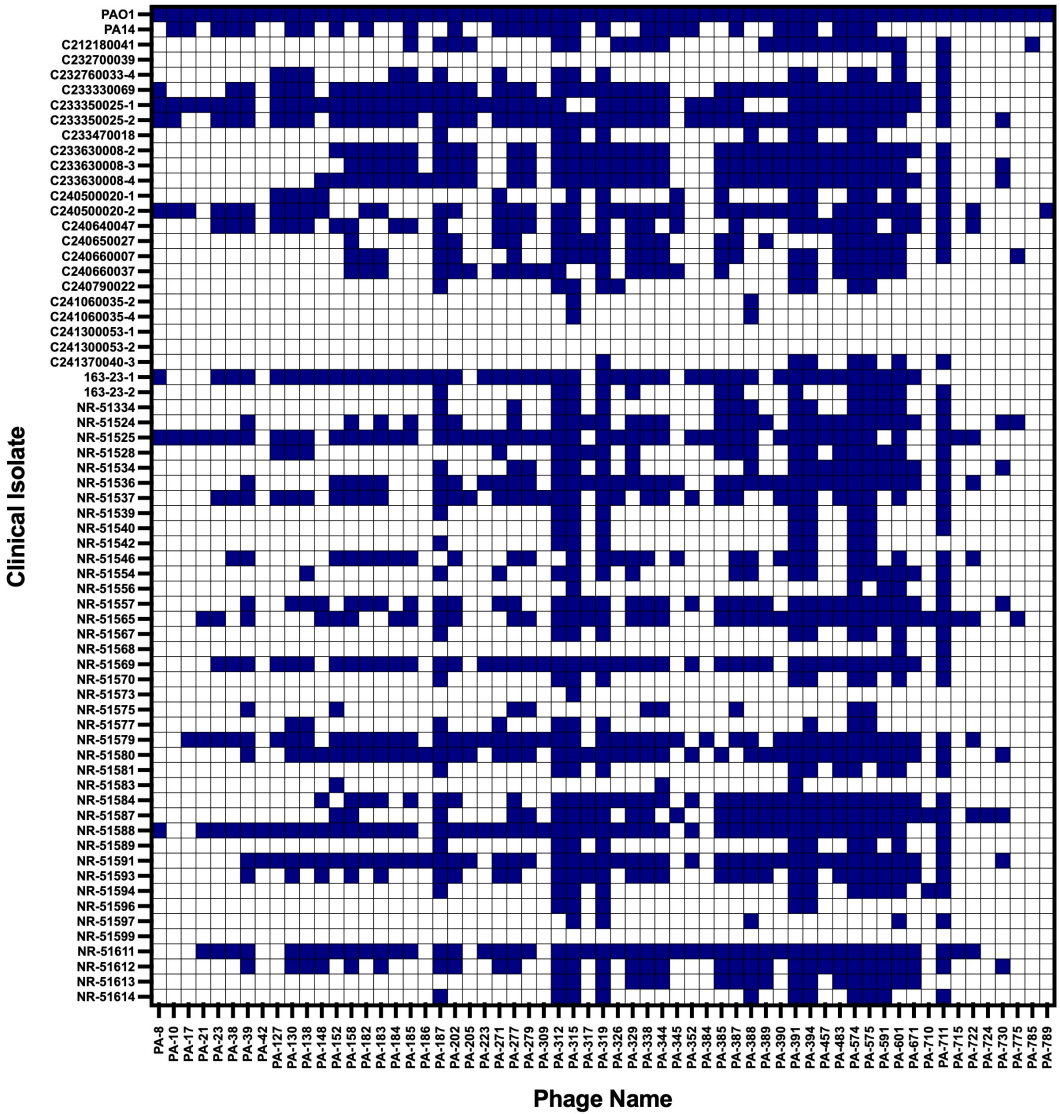
The host range of the *P. aeruginosa* phage library generated in PAO1 strain. The susceptibility of 64 clinical isolates, along with *P. aeruginosa* PA14 strain, to the 61-phage library was assessed using spot assays on double-layer agar overlays. Five microliters of each lytic phage (~10^10^ PFU) were spotted onto bacterial lawns containing ~10^8^ CFU. Lysis was monitored as clear zones of inhibition on the otherwise confluent bacterial growth. The susceptibility matrix is color-coded: blue indicates that a given isolate is susceptible to the corresponding phage, while white denotes resistance.

Eight phages (PA-312, PA-315, PA-319, PA-391, PA-394, PA-574, PA-575, and PA-711) exhibited the broadest lytic spectra, lysing between 74% and 85% of the tested human and animal *P. aeruginosa* isolates including MDR strains (**Figure 1**, **Supplementary Tables 3**, **4**). Overall, additional 17 phages lysed more than 50% of isolates, and 36 phages showed activity against more than 30% of isolates. The potent activity of these phages across diverse clinical and animal-derived strains highlights their strong therapeutic potential. The selected eight phages with a broad-host-range were subsequently chosen for further characterization, including EOP determination and antibiofilm activity assays.

### Phage virulence differs markedly among antibiotic-resistant *P. aeruginosa* clinical isolates

**Figure 2:**
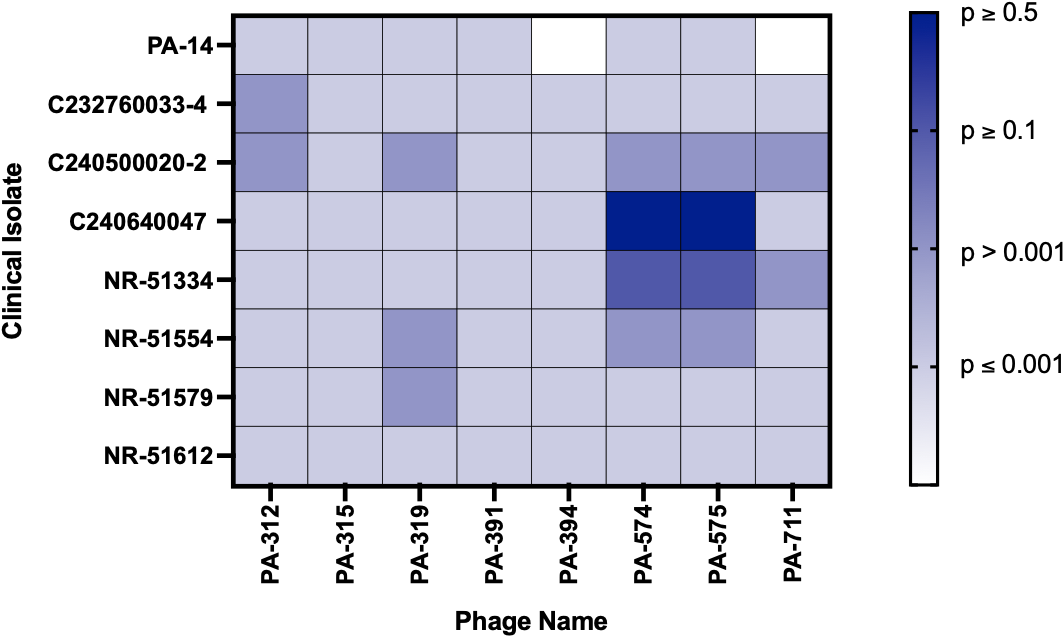
Assessment of the virulence of eight selected phages against *P. aeruginosa* clinical isolates using the efficiency of plating assay. Ten-fold serial dilutions of each phage were spot-plated onto soft agar overlays prepared with clinical isolates. Plaques formed at each dilution were recorded and compared to those on PAO1 to calculate the EOP, which reflects relative lytic activity against each clinical isolate. EOP values were categorized and color-coded as follows: non-susceptible (no lysis, white), inefficient (≤ *0.001*, light blue), low efficiency (> *0.001*, blue), medium efficiency (≥ *0.1*, dark blue), and high efficiency (≥ *0.5*, black). This analysis provides a quantitative measure of phage virulence across clinically relevant isolates and highlights differences in host susceptibility within the eight-phage library.

To further characterize the eight selected phages exhibiting broad host range activity, efficiency of plating assay was performed to assess phage virulence and lytic potency against *P. aeruginosa* clinical isolates (**Figure 2**), alongside the PAO1 reference strain from which the phages were originally isolated. The average EOP for each phage–bacterium pair was calculated as the ratio of the phage titer on the clinical isolate to that on the PAO1 host strain. Phages were categorized based on their virulence: high production (EOP ≥ 0.5), medium production (0.1 ≤ EOP < 0.5), low production (0.001 < EOP < 0.1), inefficient (EOP ≤ 0.001) and no activity (no lysis), indicating the relative efficiency of plaque formation compared to the primary propagation host (Khan Mirzaei and Nilsson, 2015). As shown in the **Figure 2**, a hypervirulent burn isolate PA14 was susceptible to six of eight phages, although all exhibited low EOP when compared to PAO1 virulence. The bovine eye isolate C232760033-4, resistant to nine antibiotics across seven classes of antimicrobials, was lysed at low efficiency only by PA-312, with all other phages ineffective. The antibiotic-susceptible canine ear isolate C240500020–2 was susceptible to selected eight phages, but lysis was low-efficiency. In contrast, the resistant canine surgical wound isolate C240640047 was highly susceptible to PA-574 and PA-575, both exhibiting high EOP relative to PAO1. The human respiratory isolate NR-51334 was moderately lysed by PA-574 and PA-575, while PA-711 showed weak activity. Another respiratory isolate, NR-51554, resistant to seven antibiotics, showed low-efficiency lysis by PA-319, PA-574, and PA-575. The human wound isolate NR-51579 was weakly lysed by PA-319, and the human sputum isolate NR-51612, resistant to a single antibiotic, was refractory to all eight phages tested. Collectively, PA-574 and PA-575 demonstrated the broadest and most potent lytic activity across the tested phage panel.

### Phages effectively inhibit early biofilm formation and prevent proliferation of viable *P. aeruginosa* in SCFM

The antibiofilm activity of eight selected phages was evaluated in SCFM by measuring biofilm biomass, as well as bacterial viability within both biofilm and planktonic populations. Biofilm biomass reduction was quantified by comparing phage-treated and untreated control groups through crystal violet staining, measured spectrophotometrically at 590 nm. Viable bacterial counts in both biofilm-associated and planktonic fractions were quantified by CFU enumeration from serial dilutions. Phage activity was compared with antibiotic treatments to assess relative efficacy. Amikacin and ciprofloxacin were included as antibiotic controls, as aminoglycosides and fluoroquinolones are commonly used for the treatment of *P. aeruginosa* infections (Zakhour et al., 2022).

**Figure 3:**
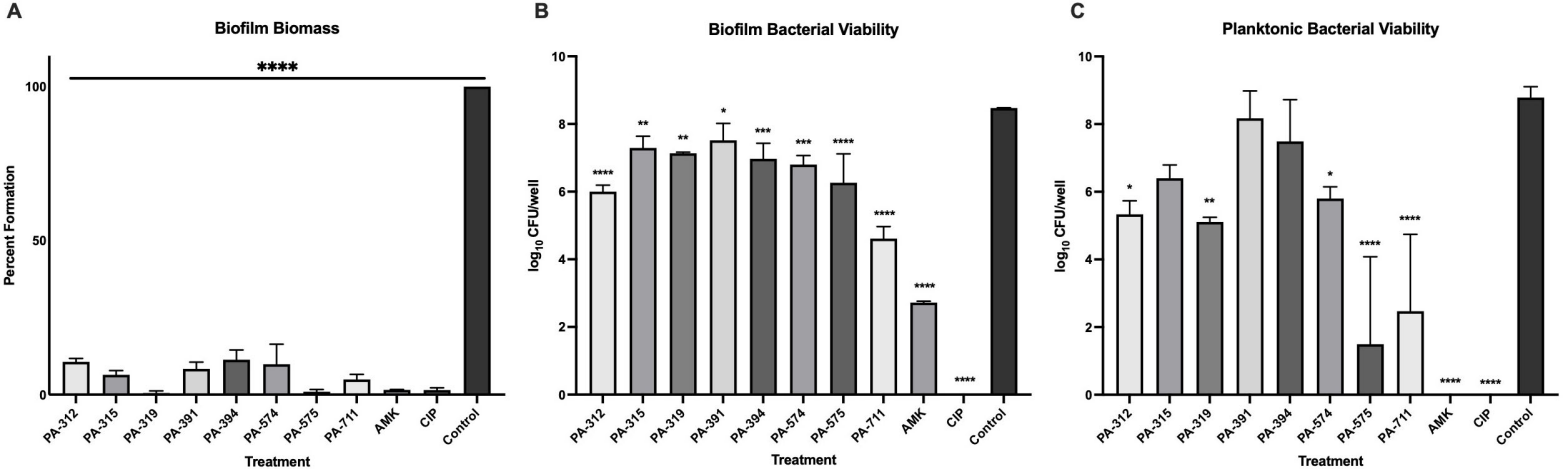
Biofilm pre-treatment assay to evaluate phage-mediated inhibition of *P. aeruginosa* biofilm formation. Phage or antibiotic treatments were applied to SCFM-containing wells of a 96-well plate at time zero, followed by inoculation with PAO1 and incubation for 24 hours. The negative control (Control) consisted of SCFM with PAO1 inoculum only. Antibiotic treatments included amikacin (AMK, 100 μg/mL) and ciprofloxacin (CIP, 50 μg/mL). Data represent the mean ± standard deviation (SD) of three experiments in eight technical replicates. Statistical significance was determined using a one-way ANOVA test, followed by a Dunnett’s *post hoc* to compare the control to the treatment groups: *p <* 0.05 (*), < 0.01 (**), *<* 0.001 (***), and < 0.0001 (****). Panels show: **(A)** Biofilm biomass quantified by crystal violet staining, **(B)** Biofilm-associated viable bacteria determined by serial dilution and CFU enumeration, and **(C)** Viable planktonic bacteria quantified by CFU enumeration of the supernatants from each well.

To assess the preventive effect of phages on biofilm development, a biofilm pre-treatment assay was performed by adding phages to 96-well round-bottom plates at time zero, followed by inoculation with *P. aeruginosa* PAO1 and incubation at 25 °C for 24 h. Crystal violet staining revealed that all phage treatments, along with antibiotic controls, significantly inhibited biofilm biomass formation (ANOVA test F = 424.0 and *p* < 0.0001; all Dunnett’s test *p-values* < 0.0001) (**Figure 3A**). Notably, biofilm biomass in all phage-treated wells was reduced to less than 12% of the untreated control. Similarly, analysis of bacterial viability demonstrated that phage pre-treatment significantly reduced the number of viable bacteria within biofilms formation (ANOVA test F = 130.7 and *p* < 0.0001) (**Figure 3B**). Each well was initially inoculated with approximately 3 × 10^6^ CFU at time zero. After 24 hours, most CFU counts exceeded this initial value, indicating that bacterial proliferation within biofilms was not completely inhibited. Although proliferation occurred, it was found that all eight phages, in addition to both antibiotics, significantly reduced CFU counts when compared with the untreated control. Among them, phages PA-312, PA-575, and PA-711 showed the strongest activity, producing highly significant reductions in bacterial viability (*p <* 0.0001). Moderate but still significant reductions were observed with phages PA-315 (*p* = 0.0069), PA-319 (*p* = 0.0020) PA-391 (*p* = *0.0364*), PA-394 (*p* = 0.0006), and PA-574 (*p* = 0.0002). Only pre-treatment with phage PA-711, amikacin, and ciprofloxacin reduced bacterial viability below the initial inoculum level. Enumeration of planktonic bacteria further clarified phage–biofilm interactions (**Figure 3C**). Phages PA-312, PA-315, PA-319, and PA-574 markedly inhibited bacterial proliferation, whereas PA-575 and PA-711 maintained bacterial counts near the initial inoculum (~3 × 10^6^ CFU). In contrast, amikacin and ciprofloxacin completely eradicated planktonic bacteria associated with biofilms. Overall, phage pre-treatment significantly reduced both biofilm biomass and bacterial viability compared with untreated controls, demonstrating that all eight phages effectively inhibit early biofilm formation and limit *P. aeruginosa* proliferation in SCFM.

### Phage treatment of established biofilms effectively reduces biofilm biomass and bacterial burden

**Figure 4:**
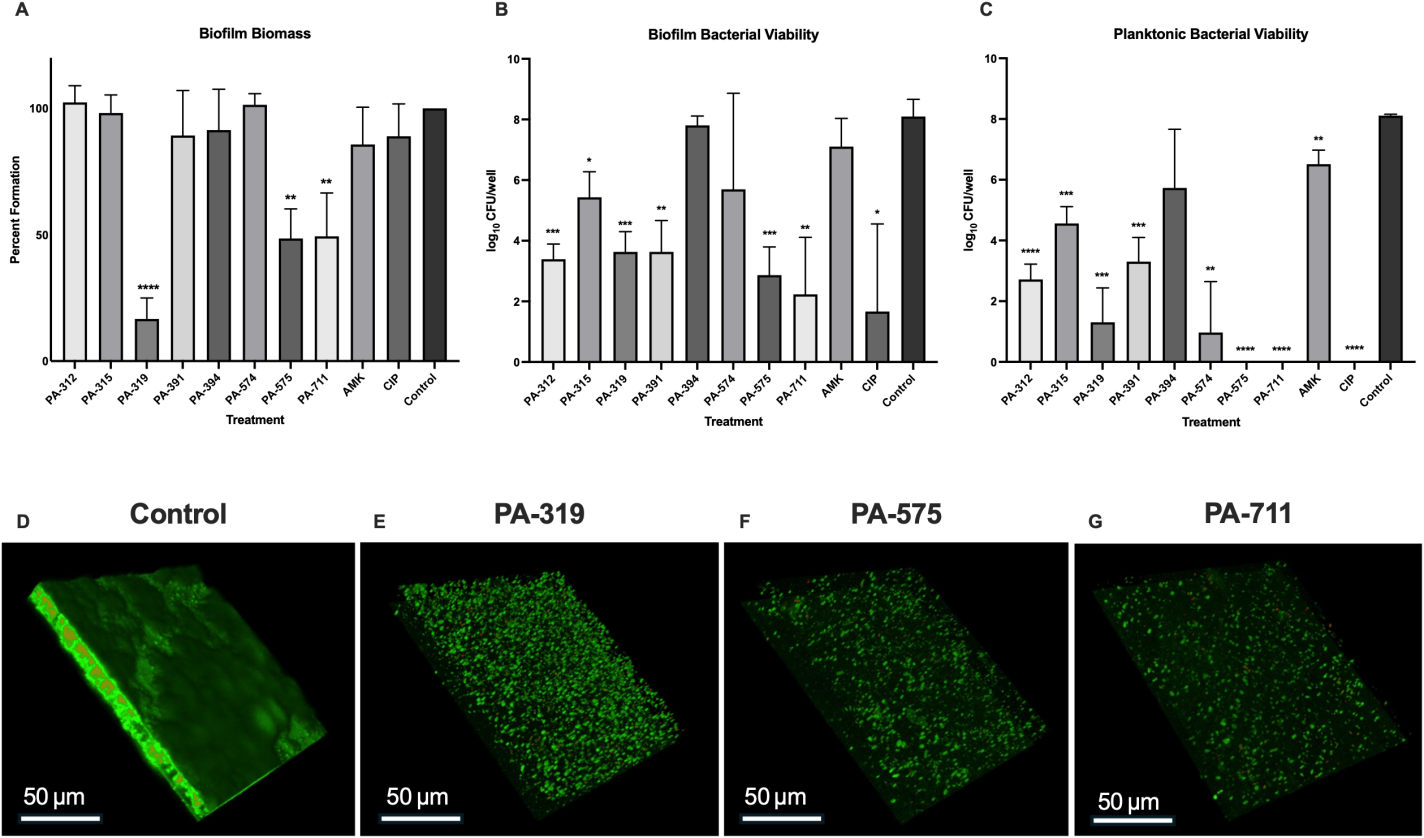
The biofilm post-treatment assay indicates that phage exposure compromises the integrity of the *P. aeruginosa* biofilm matrix. *P. aeruginosa* PAO1 biofilms were established in SCFM for 24 h, followed by treatment with individual phages, antibiotics, or SCFM alone (Control) for an additional 24 h. Amikacin (AMK, 100 μg/mL) and ciprofloxacin (CIP, 50 μg/mL) were included as clinically relevant antibiotic controls. Statistical significance was determined using one-way ANOVA tests and Dunnett’s *post hoc*, with significance levels indicated as follows: *p* < 0.05 (*), < 0.01 (**), < 0.001 (***), and < 0.0001 (****). Data represent the mean ± SD of three biological replicates with eight technical replicates each. **(A)** Biofilm biomass quantified by crystal violet staining. **(B)** Biofilm-associated bacterial viability determined by serial dilution and CFU enumeration. **(C)** Viable planktonic bacteria quantified by CFU enumeration of the supernatants from each well. **(D–G)** Confocal scanning laser microscopy visualization of LIVE/DEAD-stained PAO1 biofilms. Biofilms were established and treated as described in the materials and methods section. Viable cells are stained green and dead cells in red. Representative z-stack compilations are shown.

The efficacy of phages against established *P. aeruginosa* biofilms was assessed using post-treatment assays in which 24 h biofilms were first established and subsequently treated with phages or clinically relevant antibiotics for an additional 24 h. Crystal violet quantification of biofilm biomass revealed that post-treatment with phage PA-319 significantly reduced biofilm formation to 16.7% of the untreated control (*p* < 0.0001) with markedly different effects compared to other treatments (ANOVA test F = 17.08 and *p* < 0.0001) (**Figure 4A**). Bacterial viability assays performed 48 h after biofilm establishment demonstrated that two phages, PA-312 (*p* = 0.0094) and PA-391 (*p* = 0.0145), significantly reduced biofilm-associated bacterial counts compared to other treatments (ANOVA test F = 5.641 and *p* = 0.0004) (**Figure 4B**). Assessment of planktonic bacterial populations for these selected phages supported these findings, showing a statistically significant reduction in CFU per well (ANOVA test F = 29.46 and *p* < 0.0001) (**Figure 4C**). Notably, treatments with phages PA-575 and PA-711 completely eradicated planktonic bacteria (*p* < 0.0001), while the remaining phages produced partial but significant reductions in planktonic CFUs, consistent with their effects on biofilm-associated bacteria.

LIVE/DEAD staining combined with confocal scanning laser microscopy (CSLM) further corroborated the quantitative findings of biofilm biomass and CFU assays. Untreated control biofilms (**Figure 4D**) exhibited dense, multilayered structures composed predominantly of metabolically active cells, indicated by strong green fluorescence. In contrast, biofilms treated with phage PA-319 displayed markedly reduced thickness and density, along with fewer viable cells, consistent with the observed reductions in biomass and biofilm-associated CFUs (**Figure 4E**). Phages PA-575 and PA-711 demonstrated even more pronounced effects, achieving significant clearance of viable bacteria within the biofilms (**Figures 4F, G**). This was evidenced by minimal green fluorescence and substantial reduction in biofilm thickness, reflecting their strong bactericidal and antibiofilm activity, in agreement with CFU measurements. Overall, confocal imaging results provided visual validation of the quantitative assays, demonstrating that phages reduce both biofilm biomass and bacterial viability while altering biofilm architecture, supporting their potential as antibiofilm therapeutics.

### PA-319 exhibits strong but strain-dependent activity against clinical biofilms

**Figure 5:**
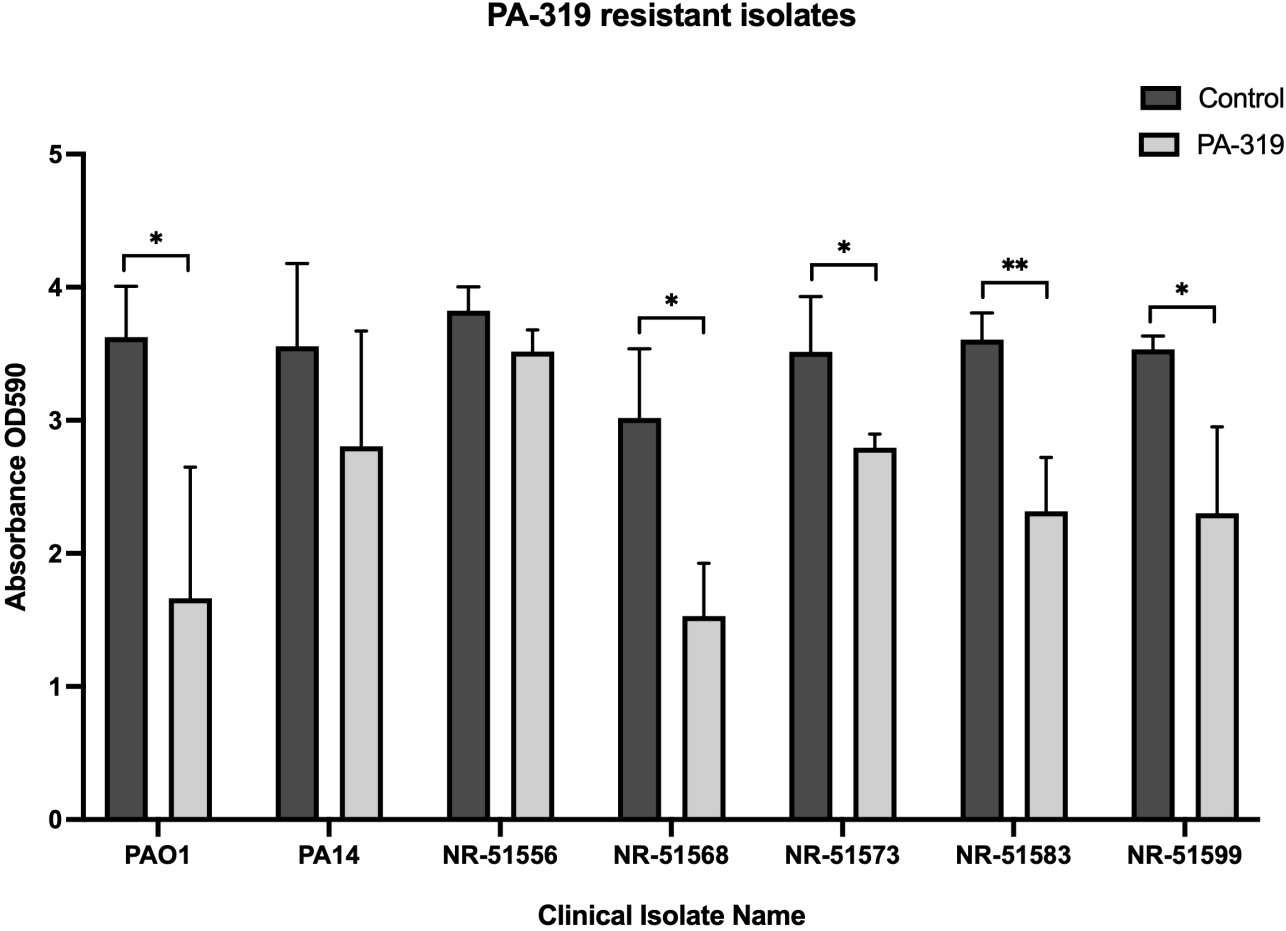
The effect of PA-319 on established biofilms of phage-resistant clinical isolates. Biofilms of *P. aeruginosa* PAO1, PA14, and five phage-resistant clinical isolates (NR-51556, NR-51569, NR-51573, NR-51583, NR-51599) were established in SCFM for 48 hours. Mature biofilms were then treated with PA-319 or left without treatment (control) for 24 hours. Biofilm biomass reduction was quantified, and statistical significance was determined using unpaired *t*-tests. *p < *0.05* and **p < *0.01* denote statistical significance between control and phage treated groups. The data is presented as the mean ± SD of three experiments in twelve technical replicates.

To assess whether phage PA-319 possesses broad-range antibiofilm activity, we tested it against clinical *P. aeruginosa* isolates, including strains previously resistant to PA-319 infection, alongside PAO1 and PA14 reference controls. The biofilms were established in SCFM for 48 h with human isolates NR-51556, NR-51569, NR-51573, NR-51583, and NR-51599, followed by treatment with PA-319 or SCFM (control) for an additional 24 h. Biofilm biomass was then quantified using crystal violet staining. Biological replicates were averaged, and unpaired t-tests were used to compare untreated and phage-treated 72 h biofilms (**Figure 5**). PA-319 treatment significantly reduced biofilm biomass of PAO1, NR-51569, NR-51573, NR-51583, and NR-51599 mature biofilms relative to their untreated controls. In contrast, PA-319 had no significant effect on biofilms of PA14 and NR-51556 at 72 h time-point. These results indicate that the antibiofilm activity of PA-319 varies across *P. aeruginosa* isolates and appears to be independent of the isolate’s susceptibility to PA-319 infection, likely also influenced by the maturity and density of the established biofilms.

**Figure 6:**
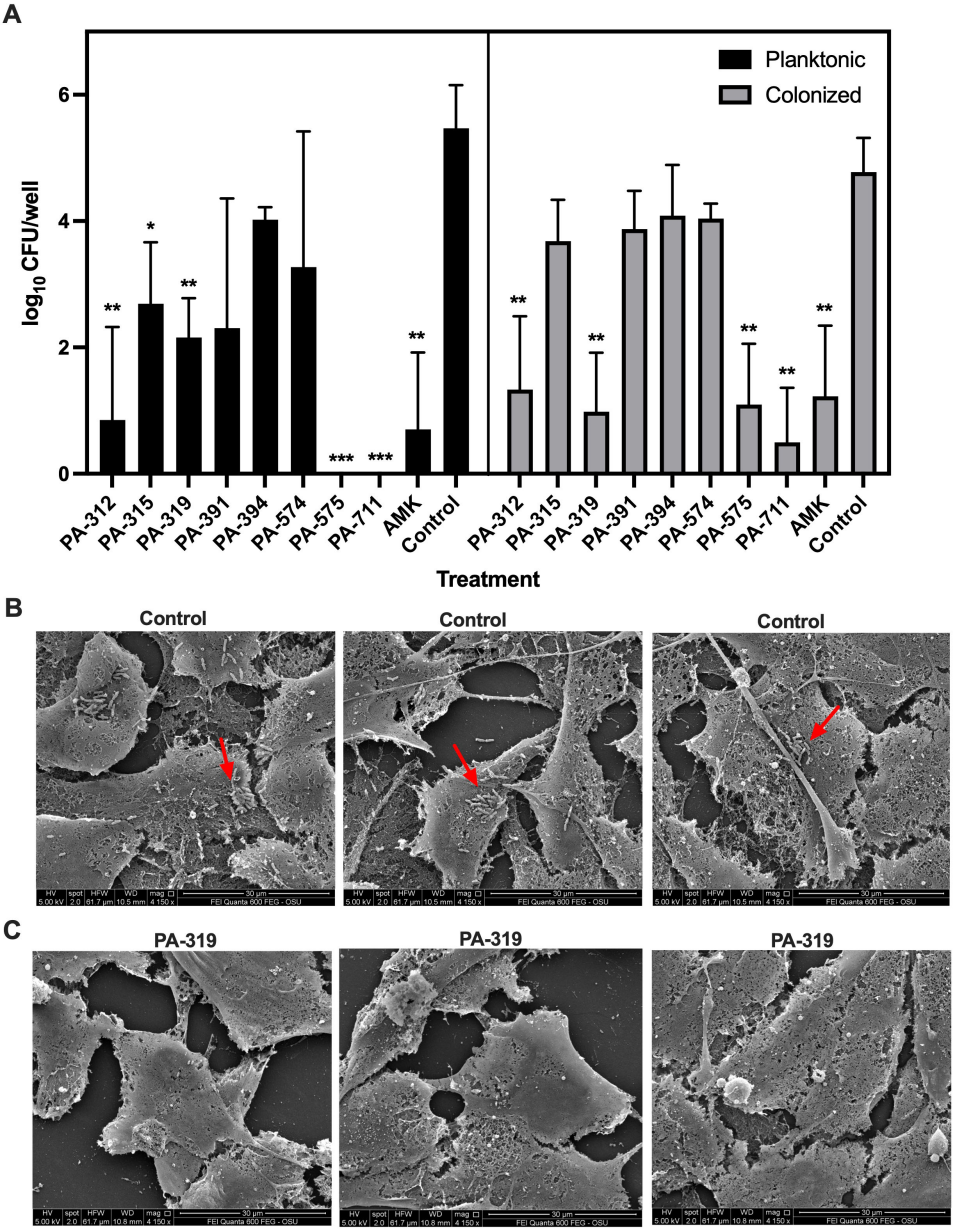
Phage pretreatment inhibits *P. aeruginosa* PAO1 colonization of human lung epithelial cells. A549 cell monolayers were pretreated with phage PA-319 (10^9^ PFU/mL) for 30 minutes, as described in the Materials and Methods section, and subsequently infected with *P. aeruginosa* PAO1 (10^6^ CFU/mL) for 6 hours. **(A)** Quantification of planktonic and cell-associated (attached and intracellular) bacteria by CFU enumeration. Data represents the mean ± SD of three experiments in eight technical replicates. Statistical significance was determined using unpaired *t*-tests between control and treatment groups (*p < 0.05, **p < 0.01, ***p < *0.001*). **(B)** SEM of PAO1 colonization on untreated A549 cells (three representative images from the control group). **(C)** SEM of PAO1 colonization on A549 cells pretreated with phage PA-319 (three representative images from the phage-treated group). Bacterial microaggregates in the colonization sites are indicated by red arrows.

Phage pretreatment prevents *P. aeruginosa* colonization of pulmonary epithelial cells. The ability of phages to prevent *P. aeruginosa* PAO1 colonization of A549 human pulmonary epithelial cells was evaluated by quantifying both planktonic and cell-associated bacteria via CFU enumeration (**Figure 6A**). Cell-associated bacteria were defined as bacterium which were either adhered to the cell surface or intracellular. Pretreatment with phages PA-312, PA-319, PA-575, and PA-711 significantly reduced both planktonic bacterial loads and colonization of epithelial cells compared to the untreated control, highlighting their potent antibacterial activity and therapeutic potential. Given its strong antibiofilm activity and marked reduction in colonized bacteria, PA-319 was selected for detailed visualization by scanning electron microscopy. SEM images revealed widespread colonization of untreated epithelial cells, with PAO1 microaggregates attached on the cell surface (**Figures 6B**). In contrast, PA-319 pretreated epithelial cells displayed minimal bacterial attachment, with only sparse individual cells observed (**Figures 6C**), consistent with the CFU data, demonstrating that phage pretreatment effectively inhibits early bacterial adhesion and colonization of pulmonary epithelial cells while exerting both bactericidal and anti-colonization effects.

### Phage genome analysis identifies jumbo phages

**Figure 7:**
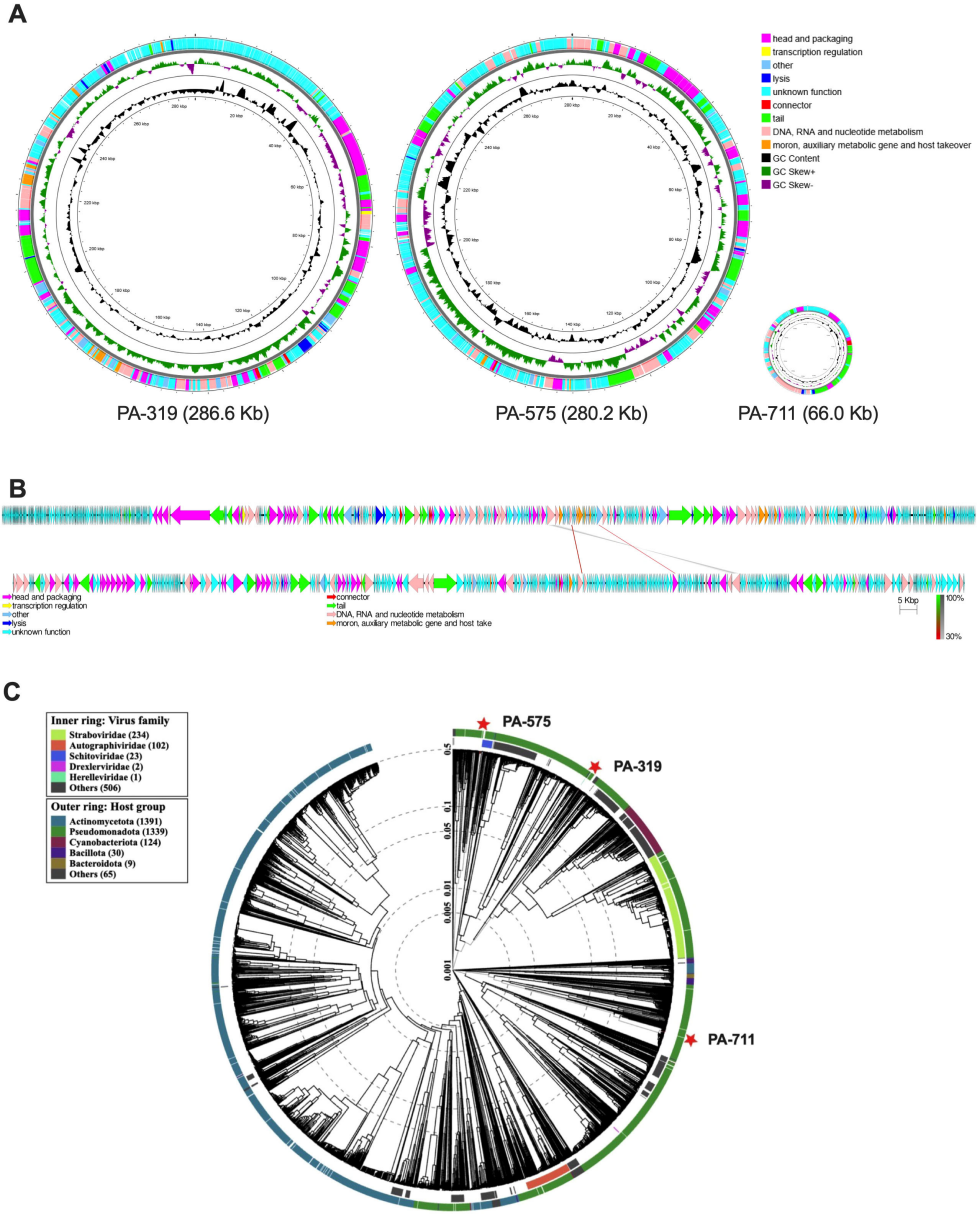
Genetic and physical maps of selected *P. aeruginosa* phages. **(A)** Whole-genome maps of phages PA-319, PA-575, and PA-711 were generated using Proksee and scaled according to relative genome size. **(B)** Local *t*BLAST*x* alignment between jumbo phages PA-319 and PA-575 visualized with EasyFig v2.2.5, with PA-319 displayed above PA-575 in the linear comparison. **(C)** Phylogenetic relationship of the three *P. aeruginosa* phages generated using VipTree based on whole annotated genome sequences. The heatmap depicts seven short regions of similarity (45–111 amino acids) identified among conserved proteins: ThyA, ATP-dependent protease/AAA-type ATPase, NrdB, and four regions in NrdA.

Assembled phage genomes were annotated using SPHAE and InterProScan and visualized with genetic and physical maps generated in Proksee (**Figure 7A**). Phage PA-319 has a genome size of 286.6 Kb, a characteristic size for jumbo phages, and encodes 537 predicted CDS, of which 391 are annotated as hypothetical proteins, with a GC content of 33%. PA-319 lacks integrases, transposases, AMR genes, CRISPR spacers, and known defense genes but encodes recombinase. PA-319 has high sequence identity (99%) to well characterized jumbo phage, PA5oct (GenBank Accession MK797984). Like Pa5oct, PA-319 lacks the machinery essential to the formation of phage nuclei within the host (Lood et al., 2020).

Genome sequencing identified phage PA-575 as another jumbo phage, with a genome size of 280.2 Kb, encoding 414 CDS, of which 298 are hypothetical proteins, and a GC content of 37%. Recombinases were identified, but no integrases, transposases, AMR genes, virulence factors, CRISPR spacers, or defense genes were found. PA-575 shares 98% identity with *P. aeruginosa* phage OMKO1 (GenBank Accession ON631220.1), a phage known for antibiotic synergy and targeting the OprM component of the multidrug efflux pumps MexAB and MexXY (Chan et al., 2016). Notably, PA-575 encodes the chimallin (ChmA) and a tubulin-like protein (PhuZ) proteins, the main component of phage nucleus-like structures (Harding et al., 2023).

PA-711 has a genome size of 66.0 Kb, with 99 CDS, 55 hypothetical proteins, and a GC content of 55%. PA-711 lacks integrases, recombinases, transposases, AMR genes, virulence factors, CRISPR spacers, or defense genes, and shares 97% sequence identity with PA19 (GenBank Accession OP831167), a characterized bacteriophage with antibiofilm activity (Alipour-Khezri et al., 2024).

A comparative genomic analysis using BLASTn revealed no significant similarity among PA-319, PA-575, and PA-711. Jumbo phages PA-319 and PA-575 were further compared using local tBLASTx and visualized with EasyFig v2.2.5 (Camus et al., 2021) (**Figure 7B**). Seven short regions of similarity (45–111 amino acids) were identified in conserved proteins, including thymidylate synthase (ThyA), ATP-dependent protease/AAA-type ATPase, ribonucleoside-diphosphate reductase small subunit, class Ia (NrdB), and four regions in the RNR large subunit, class Ia (NrdA). These findings support the phylogenetic analysis (**Figure 7C**), showing that the phages are distantly related.

### Putative depolymerases in PA-319

The ability of phage PA-319 to degrade 24 h and 48 h *P. aeruginosa* PAO1 biofilms, as well as mature biofilms formed by phage-resistant clinical isolates, suggests that PA-319 may encode enzymes capable of degrading biofilm matrices. Phage depolymerases are most associated with tail spike or tail fiber proteins. To investigate this, the predicted tail proteins of PA-319 (**Supplementary Tables 5**, **6**) were queried against known phage depolymerases (Knecht et al., 2020), but no significant sequence homology was identified. Subsequently, the depolymerase prediction tool DePP (Magill and Skvortsov, 2023) was used to analyze the genome, revealing ten candidate open reading frames (ORFs) with depolymerase probabilities ranging from 13.2% to 95.5% (**Supplementary Table 4**). Notably, three large ORFs, comprising 1319, 879, and 532 amino acids, produced the highest probabilities (95.5%, 90.3%, and 95.4%, respectively) representing strong candidates for enzymatic depolymerase activity. These findings provide a genomic basis for the broad antibiofilm activity of PA-319 and identify key ORFs for further functional characterization.

**Figure 8:**
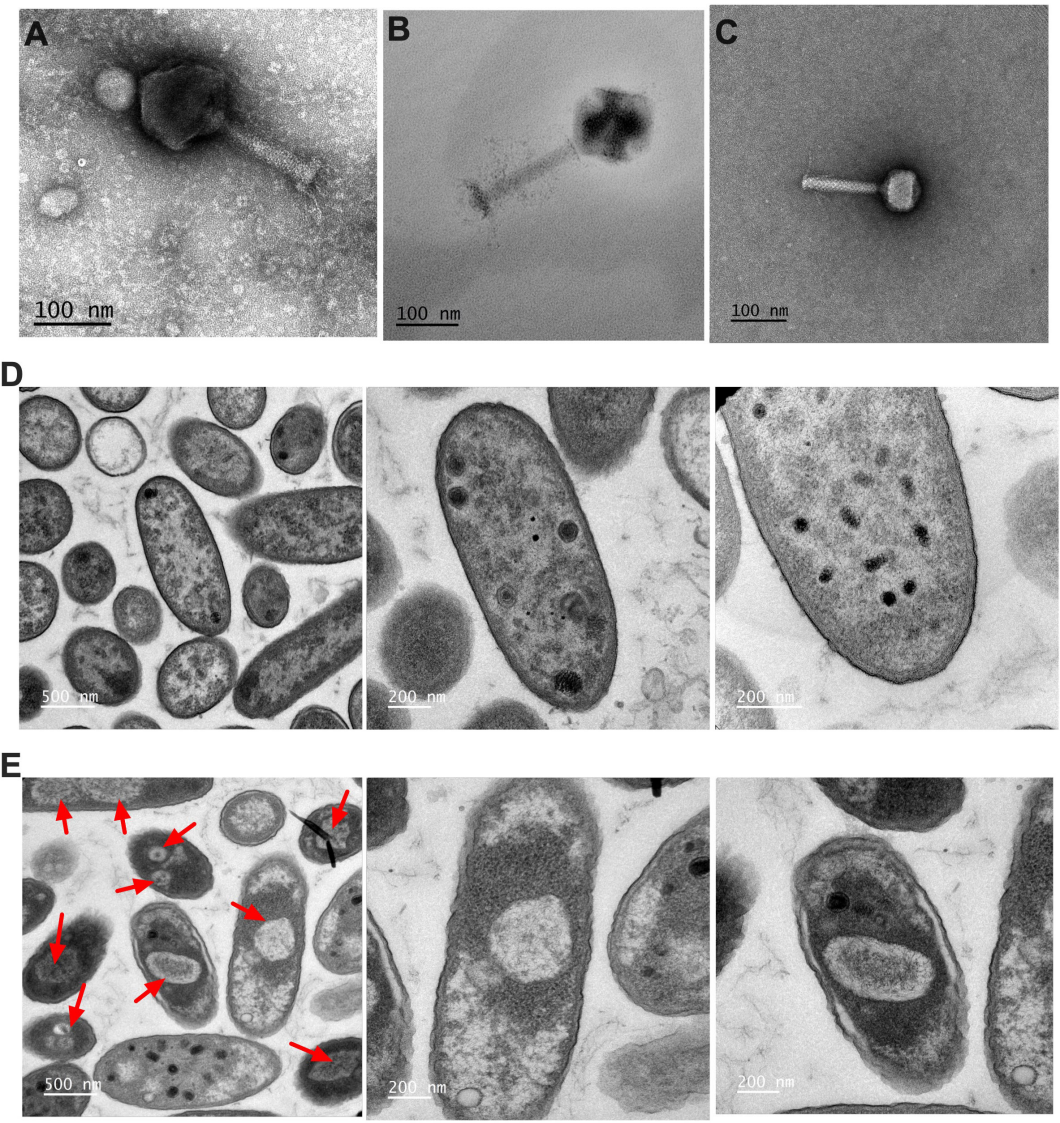
Visualization of jumbo phages and phage-derived nucleus-like structures with TEM. **(A)** TEM images compare the morphology of the non-jumbo phage PA-711 with jumbo phages PA-319 and PA-575, illustrating the larger capsid and more complex tail structure characteristic of jumbo phages. **(B, C)** TEM micrographs of *P. aeruginosa* PAO1 cells infected with jumbo phages show distinct intracellular organization. PAO1 cells infected with PA-575 display a well-defined, nucleus-like compartment containing phage DNA, whereas PA-319–infected cells lack such compartmentalization.

Jumbo phages form nucleus-like structures during infection. Transmission electron microscopy was used to characterize phage morphology, compare capsid sizes of jumbo phages (PA-319 and PA-575) to the non-jumbo phage PA-711, and assess the nucleus-forming capabilities of jumbo phages during PAO1 infection (**Figure 8**). TEM imaging confirmed the large size of the jumbo phages (PA-319: 128 ± 4 nm capsid, 137 ± 4 nm tail; PA-575: 130 ± 2 nm capsid, 187 ± 4nm tail) compared to PA-711 (78 ± 4 nm capsid, 151 ± 2 nm tail) (**Figure 8A**). During infection of *P. aeruginosa* PAO1, TEM revealed the formation of nucleus-like structures in cells infected by PA-575, whereas no such structures were observed in cells infected with PA-319 (**Figures 8B, C**). These observations suggest structural and replication strategy differences between the two jumbo phages.

## Discussion

Biofilm formation is a central feature of *Pseudomonas aeruginosa* pathogenesis and is particularly critical in the chronic pulmonary infections that develop in individuals with underlying lung conditions such as CF (Camus et al., 2021). Within the viscous and inflamed mucosal environment of the CF lung, *P. aeruginosa* readily establishes biofilms that provide both physical protection and a platform for rapid genetic adaptation. These biofilm-associated bacterial communities exhibit markedly increased tolerance to antibiotics and host immune responses, enabling long-term persistence and recurrent infection. The growing prevalence of MDR *P. aeruginosa* strains in CF care has intensified the urgency of developing alternative therapeutic strategies capable of targeting biofilm-associated bacteria (Ambreetha and Singh, 2023).

Bacteriophages have re-emerged as promising candidates for the treatment of refractory *P. aeruginosa* infections due to their ability to selectively infect bacterial cells, self-amplify at the site of infection, and be tailored to specific strains (Lin et al., 2017). Despite these advantages, key knowledge gaps continue to limit the robustness and predictability of phage therapy. Challenges include the narrow host range of individual phages, potential for phage resistance, variable interactions with the immune system, and the lack of established regulatory frameworks for clinical implementation. Comprehensive isolation and characterization of diverse phage libraries is therefore essential for identifying phages with broad host range, strong lytic activity, and enhanced antibiofilm properties.

In this study, we isolated and characterized phages with therapeutic potential against *P. aeruginosa* biofilms in a CF-relevant infection model. Sixty-one phages infecting the reference strain PAO1 were isolated, purified, and propagated. Because broad host range is a desirable trait for clinical use (Myers et al., 2023), these phages were subsequently screened against 64 genetically and phenotypically diverse *P. aeruginosa* clinical isolates, representing varied anatomical origins and antibiotic resistance profiles. From this screen, eight phages demonstrated broad lytic activity, lysing 75–86% of the clinical isolates, and were selected for further characterization based on their therapeutic promise. Notably, many of the susceptible isolates displayed resistance to commonly used anti-pseudomonal antibiotics, including amikacin, tobramycin, ciprofloxacin, aztreonam, and levofloxacin, reinforcing the potential value of phages as alternative or adjunctive agents in drug-resistant infections. Phage virulence was evaluated using efficiency of plating assays comparing titers on PAO1 and clinical isolates. PA-574 and PA-575 exhibited consistently high EOP values, indicating strong lytic activity across strains and suggesting that these phages may be effective at lower doses *in vivo.*

Because biofilm formation is a major driver of persistence in *P. aeruginosa* infections, we next examined the antibiofilm activity of the eight selected phages using complementary prevention (pre-treatment) and disruption (post-treatment) assays in SCFM, which models the mucus-rich CF lung environment (Palmer et al., 2007). All phages were tested at titers consistent with those used in ongoing clinical trials (National Institute of Allergy and Infectious Diseases (NIAID), 2025). In early biofilm formation assays, all eight phages significantly reduced biomass accumulation, demonstrating potent activity against biofilm initiation. Phages PA-312, PA-574, PA-575, and PA-711 were particularly effective, inhibiting both planktonic growth and surface-associated biofilm development. While ciprofloxacin completely eradicated bacterial populations in SCFM at concentrations far above the bactericidal threshold (50 µg/mL), both PA-711 and amikacin, even at levels well above their bactericidal concentration (100 µg/mL), substantially reduced viable cells but still allowed survival of a residual population. In contrast, the phage treatment of established biofilms revealed distinct phage-specific phenotypes. Among the panel, PA-319, PA-575, and PA-711 showed the strongest antibiofilm effects, reducing established PAO1 biofilm biomass by 83%, 57%, and 51%, respectively, and markedly decreasing the number of viable biofilm-associated cells. Confocal imaging corroborated these findings, revealing extensive degradation of the biofilm matrix and pronounced structural collapse following PA-319 treatment. CSLM z-stack analysis further showed that phage activity extended beyond the biofilm surface, penetrating deeper layers and disrupting the three-dimensional architecture. Together, these observations demonstrate that select phages can compromise both biofilm integrity and bacterial viability, confirming their potential to effectively target established *P. aeruginosa* biofilms.

This pattern is characteristic of phages that encode depolymerase enzymes capable of degrading extracellular polymeric substances (EPS), and genomic analysis of PA-319 identified putative depolymerase domains within tail-associated proteins. Consistent with this, PA-319 significantly degraded biofilms not only in PAO1 but also in four of five phage-resistant clinical isolates, indicating activity that extends beyond laboratory strains. Phage-associated depolymerases have the potential to disrupt biofilm structure, thereby enhancing antibiotic penetration, immune clearance, and bacterial dispersal. More broadly, phages are known to interfere with biofilms through several mechanisms, including direct bacterial lysis, inhibition of bacterial growth, suppression of quorum sensing, reduced production of matrix components, and enzymatic degradation of EPS (Moreau-Marquis et al., 2008). However, while depolymerase-encoding phages represent a promising strategy for treating persistent biofilm-associated infections, these enzymes have not yet progressed to clinical trials, and further biochemical and functional characterization is needed to establish their therapeutic utility (Guo et al., 2023).

Initial adherence of *P. aeruginosa* represents a pivotal step in the development of chronic infection, especially in the lungs of patients with persistent inflammatory airway diseases such as CF (Garcia-Clemente et al., 2020). As infection progresses, *P. aeruginosa* often transitions to mucoid and biofilm-associated phenotypes that are strongly linked to clinical decline (Mayer-Hamblett et al., 2014). In our study, pretreatment of pulmonary epithelial cell monolayers with the antibiofilm phages PA-319, PA-575, and PA-711 significantly reduced PAO1 colonization and decreased planktonic bacterial burden. These results parallel our biofilm experiments and indicate that these phages effectively prevent *P. aeruginosa* attachment and early surface-associated growth on both abiotic surfaces and host tissues. Scanning electron microscopy further supported this observation, showing that PA-319 pretreatment nearly eliminated *P. aeruginosa* adhesion and prevented the formation of multicellular aggregates on epithelial surfaces. This is notable because aggregate formation is a key pathogenic interaction that promotes biofilm establishment, persistence, and potential internalization within host tissues (Lepanto et al., 2011). Together, these findings suggest that these phages may be particularly valuable during the early stages of infection to prevent *P. aeruginosa* colonization and subsequent biofilm development.

Beyond their antibiofilm and anti-colonization activity, the phages examined in this study exhibit distinct genomic and structural features that influence their infection strategies. PA-319, a 286.6 kb jumbo phage, notably lacks the ChmA and PhuZ genes, which in other jumbo phages facilitate the formation of nucleus-like structures within the host (cite). In contrast, PA-575 (280.2 kb) encodes both ChmA and PhuZ and, as visualized by TEM, forms phage nuclei that can protect its genome and evade bacterial defense systems (Harding et al., 2023). Jumbo phages, defined as phages with genomes exceeding 200 kb, are relatively rare in isolation (Harding et al., 2023). Their large genomes provide extensive coding capacity, allowing them to deploy diverse host takeover and defense evasion strategies. In particular, nucleus-forming jumbo phages can spatially organize replication within a protective compartment, shielding their DNA from host defenses, including CRISPR systems. Genomic analyses using Pharokka confirmed the presence of ChmA and PhuZ in PA-575: ChmA forms the structural scaffold of the phage nucleus, while PhuZ, a tubulin-like protein, positions the nucleus within the host cell (Harding et al., 2023). PA-319 lacks both genes, explaining the absence of visible nucleus-like structures despite its jumbo genome. Functionally, these findings underscore the diversity of jumbo phage infection strategies. While both PA-319 and PA-575 possess large genomes and capsids, only PA-575 establishes a phage nucleus, likely providing replication compartmentalization and protection from host defenses. The absence of a nucleus in PA-319 suggests that its potent antibiofilm and antibacterial activity depends on alternative mechanisms, such as depolymerases and other lytic proteins, rather than nucleus-mediated replication. Collectively, TEM observations and genomic analyses reveal structural and functional heterogeneity among jumbo phages, offering insights into their infection dynamics and antibacterial potential.

This study successfully isolated a panel of sixty-one phages against *P. aeruginosa* and characterized for broad host range, virulence, and potent antibiofilm activity in a CF sputum model. Select phages from this library demonstrated strong therapeutic potential by both preventing and disrupting biofilms, with PA-319 degrading biofilm structure and PA-575 and PA-711 reducing viable bacterial populations, effectively targeting clinical isolates, including MDR strains. Collectively, our findings highlight the importance of comprehensive phage isolation and characterization for identifying candidates with broad host range, potent lytic activity, and diverse antibiofilm mechanisms. The phage library generated here provides a valuable resource for future studies, including combinatorial phage therapy, antibiotic synergy, and understanding phage interaction mechanisms that may involve modulation of quorum sensing, suppression of bacterial efflux pumps, and enhancement of innate immune responses. Continued investigation into phage-host interactions and the molecular mechanisms driving phage efficacy will be critical to optimize therapeutic strategies against chronic, biofilm-associated infections and to advance our understanding of phage biology in clinically relevant settings.

## Data Availability

The complete genome sequences of phages generated in this study have been deposited in the NCBI GenBank database under accession numbers PX884078, PX884079, and PX884080.
