# Supplementary material for: Phage isolation and functional characterization reveal strong antibiofilm activity against Pseudomonas aeruginosa in a cystic fibrosis sputum model
Source: Front Cell Infect Microbiol. 2026 Feb 13;16:1753740. doi: 10.3389/fcimb.2026.1753740 (PMC12947390; doi:10.3389/fcimb.2026.1753740)
Supplement: Supplementary file 1 [file Table1.docx]

**Supplemental Table 1.** Distribution of antimicrobial susceptibility among human-origin P. aeruginosa isolates.

| **Organism** | **Aminoglycosides** | | | **B-Lactams** | | | | | | **Quinolones** | |
| --- | --- | --- | --- | --- | --- | --- | --- | --- | --- | --- | --- |
|  | AMK | GEN | TBM | ATM | FEP | CAZ | IPM | PIP | MPM | CIP | LFX |
| NR-51334 |  |  |  |  |  |  |  |  |  |  |  |
| NR-51534 |  |  |  |  |  |  |  |  |  |  |  |
| NR-51570 |  |  |  |  |  |  |  |  |  |  |  |
| NR-51614 |  |  |  |  |  |  |  |  |  |  |  |
| NR-51525 |  |  |  |  |  |  |  |  |  |  |  |
| NR-51536 |  |  |  |  |  |  |  |  |  |  |  |
| NR-51537 |  |  |  |  |  |  |  |  |  |  |  |
| NR-51546 |  |  |  |  |  |  |  |  |  |  |  |
| NR-51593 |  |  |  |  |  |  |  |  |  |  |  |
| NR-51539 |  |  |  |  |  |  |  |  |  |  |  |
| NR-51524 |  |  |  |  |  |  |  |  |  |  |  |
| NR-51588 |  |  |  |  |  |  |  |  |  |  |  |
| NR-51587 |  |  |  |  |  |  |  |  |  |  |  |
| NR-51591 |  |  |  |  |  |  |  |  |  |  |  |
| NR-51583 |  |  |  |  |  |  |  |  |  |  |  |
| NR-51612 |  |  |  |  |  |  |  |  |  |  |  |
| NR-51528 |  |  |  |  |  |  |  |  |  |  |  |
| NR-51565 |  |  |  |  |  |  |  |  |  |  |  |
| NR-51567 |  |  |  |  |  |  |  |  |  |  |  |
| NR-51557 |  |  |  |  |  |  |  |  |  |  |  |
| NR-51584 |  |  |  |  |  |  |  |  |  |  |  |
| NR-51589 |  |  |  |  |  |  |  |  |  |  |  |
| NR-51569 |  |  |  |  |  |  |  |  |  |  |  |
| NR-51580 |  |  |  |  |  |  |  |  |  |  |  |
| NR-51573 |  |  |  |  |  |  |  |  |  |  |  |
| NR-51599 |  |  |  |  |  |  |  |  |  |  |  |
| NR-51613 |  |  |  |  |  |  |  |  |  |  |  |
| NR-51594 |  |  |  |  |  |  |  |  |  |  |  |
| NR-51597 |  |  |  |  |  |  |  |  |  |  |  |
| NR-51542 |  |  |  |  |  |  |  |  |  |  |  |
| NR-51577 |  |  |  |  |  |  |  |  |  |  |  |
| NR-51579 |  |  |  |  |  |  |  |  |  |  |  |
| NR-51611 |  |  |  |  |  |  |  |  |  |  |  |
| NR-51556 |  |  |  |  |  |  |  |  |  |  |  |
| NR-51554 |  |  |  |  |  |  |  |  |  |  |  |
| NR-51581 |  |  |  |  |  |  |  |  |  |  |  |
| NR-51540 |  |  |  |  |  |  |  |  |  |  |  |
| NR-51568 |  |  |  |  |  |  |  |  |  |  |  |
| NR-51575 |  |  |  |  |  |  |  |  |  |  |  |
| NR-51596 |  |  |  |  |  |  |  |  |  |  |  |

*P. aeruginosa* antibiotic susceptibility profiles of human clinical isolates were obtained from BEI Resources. Susceptibility results are color-coded as follows: resistant (red), intermediate (yellow), and susceptible (green). Antibiotic abbreviations: amikacin (AMK), gentamicin (GEN), tobramycin (TBM), aztreonam (ATM), cefepime (FEP), ceftazidime (CAZ), imipenem (IPM), piperacillin (PIP), meropenem (MPM), ciprofloxacin (CIP), and levofloxacin (LFX).

## **Supplemental Table 2.** Distribution of antimicrobial susceptibility among animal-origin P. aeruginosa isolates.

|  | **Aminoglycosides** | | | **Amphenicol** | | **B-Lactam** | **Cephalosporins** | | | | | | **Diaminopyrimidine** | **Macrolide** | | **Penicillins** | | | | **Quinolones** | | | | | **Sulfonamide** | **Tetracycline** | |  |
| --- | --- | --- | --- | --- | --- | --- | --- | --- | --- | --- | --- | --- | --- | --- | --- | --- | --- | --- | --- | --- | --- | --- | --- | --- | --- | --- | --- | --- |
| **Organism** | AMK | GEN | TBM | CHL | FFC | IMP | CFZ | CFV | CPD | CAZ | CTF | LEX | TMP | ERY | TUL | AMP | AMX | PenG | PIP | CIP | ENR | MBF | ORB | PDX | SFZ | DOX | TET |  |
| C233630008-2 |  |  |  |  |  |  |  |  |  |  |  |  |  |  |  |  |  |  |  |  |  |  |  |  |  |  |  |  |
| C233630008-3 |  |  |  |  |  |  |  |  |  |  |  |  |  |  |  |  |  |  |  |  |  |  |  |  |  |  |  |  |
| C233630008-4 |  |  |  |  |  |  |  |  |  |  |  |  |  |  |  |  |  |  |  |  |  |  |  |  |  |  |  |  |
| C240500020-1 |  |  |  |  |  |  |  |  |  |  |  |  |  |  |  |  |  |  |  |  |  |  |  |  |  |  |  |  |
| C240500020-2 |  |  |  |  |  |  |  |  |  |  |  |  |  |  |  |  |  |  |  |  |  |  |  |  |  |  |  |  |
| C232700039 |  |  |  |  |  |  |  |  |  |  |  |  |  |  |  |  |  |  |  |  |  |  |  |  |  |  |  |  |
| C232760033-4 |  |  |  |  |  |  |  |  |  |  |  |  |  |  |  |  |  |  |  |  |  |  |  |  |  |  |  |  |
| C240640047 |  |  |  |  |  |  |  |  |  |  |  |  |  |  |  |  |  |  |  |  |  |  |  |  |  |  |  |  |
| C240790022 |  |  |  |  |  |  |  |  |  |  |  |  |  |  |  |  |  |  |  |  |  |  |  |  |  |  |  |  |
| C240660037 |  |  |  |  |  |  |  |  |  |  |  |  |  |  |  |  |  |  |  |  |  |  |  |  |  |  |  |  |
| C241060035-2 |  |  |  |  |  |  |  |  |  |  |  |  |  |  |  |  |  |  |  |  |  |  |  |  |  |  |  |  |
| C241060035-4 |  |  |  |  |  |  |  |  |  |  |  |  |  |  |  |  |  |  |  |  |  |  |  |  |  |  |  |  |

Antibiotic susceptibility profiles of animal-derived clinical *P. aeruginosa* isolates were provided by the Oregon State University Carlson College of Veterinary Medicine Bacteriology Diagnostic Laboratory. Susceptibility results are color-coded as follows: resistant (R) in red, intermediate (I) in yellow, and susceptible (S) in green. Antibiotic abbreviations: amikacin (AMK), gentamicin (GEN), tobramycin (TBM), chloramphenicol (CHL), florfenicol (FFC), imipenem (IPM), cefazolin (CFZ), cefovecin (CFV), cefpodoxime (CPD), ceftazidime (CAZ), ceftiofur (CTF), cephalexin (LEX), trimethoprim (TMP), erythromycin (ERY), tulathromycin (TUL), ampicillin (AMP), amoxicillin (AMX), benzylpenicillin (PenG), piperacillin (PIP), ciprofloxacin (CIP), enrofloxacin (ENR), marbofloxacin (MBF), orbifloxacin (ORB), pradofloxacin (PDX), sulfisoxazole (SFZ), doxycycline (DOX), and tetracycline (TET).

**Supplemental Table 3.** *P. aeruginosa* isolate properties and phage susceptibility.

| **PA Isolate** | **Prophages** | **Species** | **Source** | **Phage Susceptibility** |
| --- | --- | --- | --- | --- |
| PAO1 | - | Human | Burn | 65 |
| PA14 | - | Human | Burn | 29 |
| C212180041 | - |  |  | 22 |
| C232700039 | - | Canine | Ear | 2 |
| C232760033-4 | - | Bovine | Eye | 16 |
| C233330069 | - | Equine | Gastrointestinal Fluid | 42 |
| C233350025-1 | - | Canine | Skin | 46 |
| C233350025-2 | - | Canine | Skin | 46 |
| C233470018 | + |  |  | 9 |
| C233630008-2 | + | Canine | Ear (no pigment) | 34 |
| C233630008-3 | + | Canine | Ear | 33 |
| C233630008-4 | + | Canine | Ear | 37 |
| C240500020-1 | - | Canine | Ear (no pigment) | 15 |
| C240500020-2 | + | Canine | Ear | 41 |
| C240640047 | - | Canine | Surgical | 31 |
| C240650027 | - | Canine | Lung | 21 |
| C240660007 | - | Goat | Lung | 24 |
| C240660037 | - | Canine | Ear | 24 |
| C240790022 | - | Canine | Ear | 9 |
| C241060035-2 | + | Canine | Ear | 2 |
| C241060035-4 | + | Canine | Ear | 2 |
| C241300053-1 | - | Canine | Ear | 0 |
| C241300053-2 | - | Canine | Ear | 0 |
| C241370040-3 | - | Canine | Ear | 7 |
| 163-23-1 | + |  |  | 44 |
| 163-23-2 | + |  |  | 13 |
| NR-51334 | - | Human | Leg/foot ulcer | 15 |
| NR-51524 | + | Human | Respiratory | 30 |
| NR-51525 | - | Human | Wound | 47 |
| NR-51528 | - | Human | Respiratory | 20 |
| NR-51534 | - | Human | Respiratory | 19 |
| NR-51536 | + | Human | Wound | 36 |
| NR-51537 | - | Human | Wound | 34 |
| NR-51539 | - | Human | Urine | 9 |
| NR-51540 | + | Human | Respiratory | 8 |
| NR-51542 | - | Human | Respiratory | 8 |
| NR-51546 | + | Human | Wound | 27 |
| NR-51554 | - | Human | Respiratory | 17 |
| NR-51556 | - | Human | Wound | 5 |
| NR-51557 | - | Human | Wound | 35 |
| NR-51565 | - | Human | Wound | 38 |
| NR-51567 | - | Human | Wound | 10 |
| NR-51568 | + | Human | Wound | 2 |
| NR-51569 | - | Human | Wound | 42 |
| NR-51570 | - | Human | Respiratory | 10 |
| NR-51573 | - | Human | Respiratory | 1 |
| NR-51575 | + | Human | Urine | 9 |
| NR-51577 | - | Human | Respiratory | 10 |
| NR-51579 | + | Human | Wound | 45 |
| NR-51580 | - | Human | Respiratory | 41 |
| NR-51581 | - | Human | Wound | 11 |
| NR-51583 | - | Human | Respiratory | 3 |
| NR-51584 | - | Human | Respiratory | 32 |
| NR-51587 | - | Human | Respiratory | 31 |
| NR-51588 | - | Human | Wound | 47 |
| NR-51589 | + | Human | Respiratory | 10 |
| NR-51591 | - | Human | Respiratory | 44 |
| NR-51593 | - | Human | Wound | 29 |
| NR-51594 | - | Human | Respiratory | 12 |
| NR-51596 | - | Human | Respiratory | 5 |
| NR-51597 | - | Human | Respiratory | 5 |
| NR-51599 | + | Human | Urine | 0 |
| NR-51611 | + | Human | Fluid | 47 |
| NR-51612 | - | Human | Sputum | 31 |
| NR-51613 | - | Human | Urine | 18 |
| NR-51614 | - | Human | Sputum | 11 |

Numbers represent BEI Resources identification codes for *P. aeruginosa* clinical isolates. The symbols (+) and (−) denote the presence and absence of prophages, respectively. Phage susceptibility is expressed as the number of phages (out of a total of 61) that produced clear lysis zones on the respective isolate.

**Supplemental Table 4.** Host range profiles of the eight phages of interest across sixty-five clinical P. aeruginosa isolates. Phage infectivity was evaluated using the double-layer agar assay, wherein each clinical isolate was embedded in soft agar and phages were applied by stamping onto the overlay. Productive infection was determined by plaque formation, indicating lytic activity against the corresponding isolate.

| **Phage Name** | **PA-312** | **PA-315** | **PA-319** | **PA-391** | **PA-394** | **PA-574** | **PA-575** | **PA-711** |
| --- | --- | --- | --- | --- | --- | --- | --- | --- |
| # of susceptible  PA isolates | 50 | 55 | 53 | 52 | 51 | 54 | 52 | 48 |

**Supplemental Table 5. Predicted depolymerase activities in phage PA-319.** DePP was used to predict the likelihood of depolymerase activity among all tail-associated proteins in PA-319. Gene name, size, predicted product, and annotated function were assigned based on Pharokka analysis of the PA-319 genome.

| **Gene Name** | **Gene Size (aa)** | **Gene Product** | **Gene Function** | **Probability_DePol** | **Percentage** |
| --- | --- | --- | --- | --- | --- |
| APNARBLE_CDS_0188 | 1319 | Baseplate wedge subunit | Tail | 0.9546928571 | 95 |
| APNARBLE_CDS_0189 | 130 | Baseplate wedge subunit | Tail | 0.1317989418 | 13 |
| APNARBLE_CDS_0193 | 477 | Baseplate hub subunit and tail lysozyme | Tail | 0.7646920635 | 76 |
| APNARBLE_CDS_0195 | 99 | Baseplate wedge subunit | Tail | 0.1342373016 | 13 |
| APNARBLE_CDS_0211 | 284 | Baseplate hub | Tail | 0.4456579365 | 44 |
| APNARBLE_CDS_0228 | 456 | Tail sheath | Tail | 0.8757928571 | 88 |
| APNARBLE_CDS_0232 | 532 | Tail protein | Tail | 0.9033880952 | 90 |
| APNARBLE_CDS_0261 | 250 | Head-tail adaptor Ad2 | Tail | 0.4335952381 | 43 |
| APNARBLE_CDS_0263 | 879 | Tail sheath | Tail | 0.9536936508 | 95 |
| APNARBLE_CDS_0265 | 206 | Tail tube protein | Tail | 0.3628997354 | 36 |

**Supplemental Table 6. FASTA-formatted protein sequences of tail-associated proteins in phage PA-319.** The genome of PA-319 was annotated using Pharokka, and amino acid sequences of predicted tail-associated proteins are presented in FASTA format.

| **Protein Sequence** |
| --- |
| **> APNARBLE_CDS_0188**  MSQDVRQSNLFAAEDYRKIYKSFKDVDFKAYDFDSLKTALVNYVQIHYPEDFNDYIESSEFIAIIELLAYLGTSLAFRMDLNSRENFMDTAERRDSIIRLARMINYQPKRNIPAKGLFKLIGVQTSEPVTDSQERNLNNRTVFWNDPNDIDSYDKFITILNAAFASTNPFGRPFKSGTVGNIPTSLYQLNNVKRVNVAFPIPIVVNNVALPFEVVNSDFDETFTERQPDPDDAFHVIYRNDGQGLDSANTGFFLQFKQGTLAFKDFAFDFPVQNRIIDINDRNINETDVWVQEINEIGTVEEKWTKVPNVSGGTNIIYNSINLGIRNIFEVVSRVNDQISVKFSDGNFGTIPTGTFRVWYRVSSNTTFTLRPEEASGMELVIPYIGADSQQYDLRLIFSLEQTISNSSPTETNDEIKTRAPQVYYTQDRMVNNEDYNVFPLTQGSSIAKVHAINRTHSGHSRYIDINDPTGFHQNLNIFGEDGSIYNQSTVPSTTVAMGDKLVSENIVVRNNLQTFIRNQDLINFYWNNYLSEYIRYRQNPTPNPINTSIVRPGVPNIFRYTSTQAKWVTYPSSDATNTGYLVKGSTNYIYPTNYNNRPEGEFRFLSVGSKIKFTNGTEYKEVTVKSITNPVSQGDATTDNEFIATRLNASLFVFDIEIPANWYVDSVYPRYKTTFDTEETTDIANQLFRRNEFGLKYDIDVDPEGSWFIVLGVPTTNLDNFEFDLSLSQPAVRKDWMLVLTYSTETDNYTFLSRGTKFIFETNGTVEFYYDTDETEFDINTGSAQRDEIEILGINTGPTIVENWQFIGNGQWRLVNGIVSLTYPDNKIILKYRDTLPKEIQYWQNGILLPGALPTGAASAGVLDAEYVGQQVNDIIRIFYSNQGQLGNSIVWNSFQTFIESDGYSDPRKIVVVPADRDRDGIPDNTFIFEQFVSPTDLVFHEKTTDYDGYEYNRLWQASWLDLRQQNPNSLDFNYNDVVARDLVLIASAAESSFKSLLRTKVLEAIYSDNYNSNNSVDGTTIFIERIENDGINIVTQNLDSPGSRIFETIRINNGNNQTINEITGITAAKANSSSLAQFMTLTASYAVLSYVFDDDHSVSNGRGYTQNEDSQFYPLHYKWKHYAPSDNRIDPSISNIVDMVTLTQSYYDDILKWKNQNLPIERFPESPTTEELRIQFGDLSTYKMLSDQIVFQPARFKVLFGQQAREEYKCTFKVVKVGSTTLTDNEIKSKVVSAIDEYFNINNWDFGESFYYTELAAYIHQTLAGVIGSVVIVPIEQTSKFGNLFQIRAESDELFISTATVANVEIVNNLTEMNMRI |
| **>APNARBLE_CDS_0189**  VAKVFIGFSTVGKVRPPYQVNDIDLVKVDLMNHFYTRKGERVMLPEFGSIIHDYVMDPLDEYTVGIVVDDVKDVIGSDPRVQLNSDDDIKVIQLHNGIRIEVLLTFLPYQTPERLIAIFTQENSSNIDRL |
| **>APNARBLE_CDS_0193**  MSTYSRTVGVNPSASKTPTGRAQKYYGMYIGFVKDNVDSNRMGRLRVWVPEFGTDPSDESGWLLVGYCSPFAGATNPMTIGGSQDVSAFHETQTSYGMWAVPPDLENQVAVMFANGDPAKGFWFGCIWQQQMNHMIPAVAGSVNNYETAGKSLPVGEYNKNTLEKVNAQTINKPVASLAQGLKTQGLINDNIRGVSSSSARREAPSQVFGILTPGPVIDGANARRHGGHQFVMDDAPTSEHITLRTRSGAQIRLDETNGIIYIINKLGTAWIELDNSGNVDVFGAKSGSLRFMEDFNIRADRDINIEAGRNINIKASKDYTTVSTGGIAPELTGQGGDIFIQANNNLNIQSQKDTLLNQLGGSLHNTAGVDISLKAGSNYNLQAAGQIATATNGTYGLSAGGDIIELGANIHMNGPQPPAPDSPTEASTPTTNGKTNILPEFSDANRYNRMSQPGVTTIVSRFNTFEPCPEHKNKGA |
| **>APNARBLE_CDS_0195**  MANNISSTKSRYYDTPIKDFYLDLWVPRKILPNITDELIVIAPKYDQRPDLLSYDLYGTEELWWVFAVRNMDELIDPIYDFTAGKSIYAPTIESLEGII |
| **>APNARBLE_CDS_0211**  MSNKLHQYYRTFKSYIRLPSGNSFYPSNVVEYTDSGELGVYAMTGKDEVLIKNPDALVNGEGLKEVIKSCVPGIKDVNKLLINDIHMICVAIKAASYGNTIDISSTCPHCNHKNLYGIDLLNVINTSTYLEDQYVVNLDNGLSIFIRPYDFSDNMTVAKTALEQAKFIRIMQSDMYSDEEKLSAFSNTINELSSLNFELIAKTIVRIVDETNGIDIVNNTAQTLADIKEFIANIDRADVNKIDSLLKEINQVGVNGDFDAVCDNCNKSFVVPVDFNPVSFFRES |
| **>APNARBLE_CDS_0228**  MNYFYDEQLRRYLLQFLRVFSDINIRTAPDDNGVSIQKRVPVIYGDMSRQVASIINGNNQNTVIPVPIMAGYITGLDLAPDYRVDTQGVKVSNVTELATDANGSYINKPGNRYTVEAYMPVPYKLTTRLDILTSNTDNKLQILEQILVLFNPSIQLEQNQNPLDWSRIFELELKETQWSNRTIPVGTSTDYDISSLIFQMIVYINPPAKVKKQKIINTIISNIYTDSFSDIKSTTDFFNVTPGTVKEQLCVTPGNYGLRVIDNKLTLLNTYGDVSSDSWDKLLNIYGSVEQNVTTVKLIQGNIDNPDIEIIGYIEPSSTNTTTFTPIDETLPSATIPPINRIIDPSKMFPGNKLPEAQIGQRYMVLNSLTKGEEPAIKNSYPWGNSFIAYDSDIIEYTGTQWVVTFNARVTNNNPVYVKNLYDKELYKFKDREWQYGYAGDYKAGYWRVDNICSIS |
| **> APNARBLE_CDS_0232**  MPYTIKRYSSVNGGSSKPNKTVNDIQIDNTYSSIGLIGSYNESYKSQFFQNMLWKMENFSHTIAPLNPIEGQLWFNPDEKSVKVYISNDKPTSVSSDWKNLGSTILNELTDHESRTGINDPHETTKEQVGLGNVPNVYTFNKTLNLADVLDASICRSNLEVYGTSETYNKTTVNNLFMTKGGTVNNTDRLDGLTSADFVLVDNPVVYRTFDTTSIITNTALTYNNSKGSIVVNTGAGNIELLSGIRSDGRIASANDTGAMLSFINDNSDPMVTMVVFNRATANNQTAQPVRSYRFTKTNLIIDYDTNPSEMYHTGRIPSNAAVGSLPLNGKAVNTLLLNGMYQSSSPNDNAVALRDGAGDVHVRHIQVSNPDENIETGNLILFRNSNTSDNYVRRANVSSFQSWFRANEPKGVVKAWIQFNGITGSTYKSHNCTLTKLGTGKYRIYYNSFSTPAAGSVVPFVGVTSDGQISTGMAAGTGRLSYNSSLVYLRSTTYTDLEFFRTYNINYNNYSLDSFTNVYADANNITFTWFF |
| **>APNARBLE_CDS_0261**  MATSTARTEIKKQIMRRLGNSMTDVELDPEDLDLAIDFAIDRYRQRAENACMESMLFITLQRDETEYTLPTEVQEVRRLYRRSVGSGTSGGTNFDPFESAFSNIFFLQAGRTGGLATWHLFSEYQETLGRIFGSEINFTWNYADRKLTIMRKFQGAETVLVVVWVAKSEDVLLTDVYSRPWLRDYAIAQSKYMLGEARSKFTNGLPGPNGSVTLNGDQLKQEAFAEMERLENEIINYTTGTSFGMPFIIG |
| **> APNARBLE_CDS_0263**  MATLISPGVSVTITDESFYASSGPGTVPLIIIATAQDKTDSAGTGIAPGTTRQNAGRLWLISSQRELLQTFGVPTFKTVGGTALHGYELNEYGLLAAHSYLGISNRAYVVRADVDTSQLEPSDTEPTANPADNTYWLNPTKVVSGLYESNGTDWVKANVFYATEFDAAGRPANVPSSYKYAMSVGQAAPNVENAIYRRNISGAWVLITANDNTVVYNKVYPVVGAVGNLWVNLRETSYALSRFDANVGSFVSVSTPLYKTSDDATDGFGTTLRQGSVFVQYNAKGTLSSAVNPAVTELQHKVKIHNGNTFTAVSVTLAATGPYSDTLVLGRYNGTSVTVNASGSSVEAFAQSLGQALVSNGVTDISVNYNTASRMVALVNDSGKDIIVTSSTMFSAGIYSNWVPLEYSSAVKQPSGQLDNGTLWYSTDYKVDILVNDGVGAWTDLAGSLFVQSAEPTTNVADGDVWVSTSSAGNYPVIYRRVNNQWRLVDNTDQTTPNGVIFADARPVNSGALDADAPDALAYPQGILLFNTRYSTRNVKQWKNNYTFEGNVIGDRWVSVSGLRSDGSLYTGGEAVKQVVVRSIASTIQANEDIRSENVFYNLIAAPGYPELIDELVALNVDRKQTAFVIGDSPFTLSATGTNLQEWSSNSKLATENGSDGLVTADPYLGVYYPSGLSTDLNGNNVVVPPSHMLLRTIAYNDQVAYPWFAPAGYQRGIVSNATSVGYINSEGEFVAVSLNEGQRDTLYTNNVNPISQVPNRGIVVWGQKSRNPVTSSLDRVNVARLVNYVRYQLDLLLKPFVFEPNDSRTRQAVQVAVEAFLSEIVTLRGITDFVVDCSDSNNTPDRIDRNELWVDVAIVPTRAVEFIYVPIRLQNTGS |
| **>APNARBLE_CDS_0265**  MATLDKFGVPSSAGSERGILQPKLQYKFRVSFDGFGDGSNSRQLTQSVVSVERPKINHASTAVHSYNSVAYAMGKHEWQTIEIVLRDDIQNSVVNVVGKQLQKQMNHFEQSSPVAGRNYKFTMRIESLDGNNNTAPLEYWDAEGCFLLDVNYGSRAYDSSELTLITLTVRADNYTHHAGGNEVALGGDPFTDDDAGLRGAPGARVN |
| **>APNARBLE_CDS_0266**  MAFDDIFGTIAGAAGDIFGATVGEAGNILSSVATRSAKKFLGGLIGDGHGAYLRDSRHATYNFGLRGSYIQQNYPRSKFQYLVKINYNTIDPVKSFVDTYLNRDEQDMIVPLIKRIDMPSMQIDTEKLNQYNKWRISQTKIKFQPITMVLHDVVDGKTLRFWEMYYEYYFNDGVYAKKTSDSISNFDPIRNTLRRGELNVSEFYNDIISKEGVFKRDFGFRLDTQVFNTTSFIGKYSRYLFDSIDIYQVHGQSYSRARLVNPRITDFRHDALAYDNSSDLMELTFTFEYEDVVYDNFVRPLAETELEFYDNANNLELDYQPARPPVSVRVRNDSTEGGFGRSSSGLGNVLGDITGDIFGGIRSGVSTVLDATGVNGVLDNIGVGAVLGSAANSVLGNIERNVTDYVTTFPENAARAVGESIFTGEVEFPVDPKGSMRKILDQAKSETTGTIRRTFETGVNTAITGVLNGAGKIIDNPRGSRNTNVSNRSVGL |
| **>APNARBLE_CDS_0373**  MSNLNDFFGSSGSEGGGAEVKRLDDLVDVSVASPTNGQTLVFDNTGWVNRKLSYNDLSNLPSISNTLSGLDDVDTAGTIAGYVLQFNGTKWVGMPASSSISLDDLSNVAITTPSINQTLLFNGSTWYNGSVDFNNLINTPVIPTHLGDLSDVSVSASTQEGSVLVFRTGQWIGSVINYNDLSNTPTIPQNDSFTFLGLSDTNDIPEANGFLKWNNLSTEIEYVTSIPSSAITGLANVATTGLLSNLVDVNVSGVSNGQVLTYNSSSSTWVPTTPSTGGGTSDPTAIFSTDGNTYINTEELVDELVLKTGPGSGNVNVILGGSGKIVVEGQSPEIISETSLKLTGSNLYLNELSWPTTDGTSGQVLATDGNGNLSFITVSSGGTTILITWRGEWEETSYNINDAVSYAGSSYICVAEVAGGDISIPPSSTNLNWNILALKGEDGSFFTYKGTYDSNTEYVDKDVVYYNGSSYVVLDGQGPITGITPTDQSKWGILALAGSGGTGSGFTYKNEYNNSETYQDQDVVSYGGSSYIVSPGNGPVTGILPTDSSIWGIIALKGQDGSGTGNQSLEDLTDVSSATPTSGNVLVANGSSWSSSKLSYSQLQGLPTLSVVATSGSYNDLLDKPSNTGTFIGLSDTDTPSLPNGFLKWNAQGTSISYVQSISSESISGLATVATTGDYSDLTNTPNIPVVLDDLTDVNLVTPPVTGQALVYNGTYWLAGNAGSGSSTLASLTDVNLTTPTDNQVLSYDNTQNLWINTQIDYNDILNTPSIPVNNSFSLVGLNDTDNSAIPNAFLKWNSTGNSVEYVQSISASSITGLSTVATTGDYNDLTNLPSIPENISDLNDVDTSTAPTDGQILIYSAALGKWIPGDQSGTSPSPGGFPGEWLKINYTPGNLIGTIECSPNISYTIVDNVANNNLTLDLVFANRPYPPAGWMWYGYLVTQGRYVLKMQDTTSHPHTLELTTTENPFGTGTGKLSGFQCRVSTTGAQSYGPLQPTHAYVIFGW |
